# Optimized Gradient Waveforms for Tensor‐Valued Diffusion MRI Under Time‐Dependent Diffusion Using Double‐Isotropic Matched Encoding (DIME)

**DOI:** 10.1002/mrm.70602

**Published:** 2026-09-24

**Authors:** Felix Mortensen, Viktor Olsson, Athanasios Grigoriou, Samo Lasič, Malwina Molendowska, Ronnie Wirestam, Filip Szczepankiewicz

**Affiliations:** ^1^ Department of Medical Radiation Physics Lund University Lund Sweden; ^2^ Radiomics Group Vall d'Hebron Institute of Oncology, Vall d'Hebron Barcelona Hospital Campus Barcelona Spain; ^3^ Department of Diagnostic Radiology Lund University Lund Sweden

**Keywords:** concomitant fields, diffusion acquisition, experimental design, microstructure, nerve stimulation, time‐dependent diffusion MRI

## Abstract

**Purpose:**

Spherical and linear b‐tensor encoding (STE and LTE) enables quantification of microscopic diffusion anisotropy and diffusional variance. However, such waveforms are often variably sensitive to time‐dependent diffusion, causing rotational variance and spectral mismatch. We propose double‐isotropic matched encoding (DIME), which enforces isotropy in diffusion‐ and restriction‐weighting tensors (B and M) and STE/LTE pairs matched in restriction sensitivity.

**Methods:**

DIME uses trapezoidal pulses, optimized to yield isotropic B and M while generating a spectrally matched LTE. The design ensures concomitant gradient balance and constrains peripheral and cardiac nerve stimulation. Rotational invariance and spectral matching were assessed by simulations in ideal and realistic substrates at 80 and 200 mT/m via CV across rotations and STE/LTE mean diffusivity (MD). DIME was compared to numerically optimized waveforms (NOWs).

**Results:**

DIME had the lowest rotational variance and best spectral matching. In cylinders, the median (IQR) CV was 2.0% (1.5%–3.3%) and 1.7% (1.2%–2.7%) at 80 and 200 mT/m, versus 2.5% (1.8%–4.1%) and 2.0% (1.4%–3.5%) for NOW. In spheres, the maximum MD mismatch (ΔMD) was 0.08 versus $0.52\;\mu m^{2}/\text{ms}$ for NOW. In realistic substrates, DIME was superior (ΔMD = 0.01±0.01) compared to NOW ($-0.16\pm 0.10\;\mu m^{2}/\text{ms}$). DIME increased encoding duration by 13–22 ms.

**Conclusion:**

By controlling both B and M, DIME improves STE rotational invariance and reduces spectral mismatch under time‐dependent diffusion, enabling more reliable tensor‐valued encoding tailored to hardware and nerve stimulation constraints.

## 1 Introduction

Diffusion‐weighted magnetic resonance imaging (dMRI) probes the random motion of water molecules in biological tissues. This has provided a wealth of contrasts sensitive to microstructural features such as cell size, shape, orientation, and permeability [1, 2], with a wide range of clinical and research applications [3, 4, 5, 6, 7].

The sensitivity to microstructural features arises from the application of time‐varying magnetic field gradients, or gradient waveforms, that link the diffusion process to the MR signal [8]. Conventional diffusion encoding along a single direction per shot is often described by the strength and direction of the encoding (b‐value and b‐vector). More generally, diffusion encoding along multiple directions within a single shot is described by a b‐tensor, which is sufficient to determine the diffusion‐weighted signal when the diffusion in the underlying tissue is a mixture of Gaussian processes [9]. The benefit of tensor‐valued diffusion encoding is that it generalizes earlier double diffusion encoding concepts [10, 11] by introducing an additional encoding dimension through the shape of the b‐tensor, thereby modulating the sensitivity to microscopic diffusion anisotropy [9, 12]. For example, q‐space trajectory imaging (QTI) [9] commonly combines linear and spherical b‐tensor encoding (LTE and STE) to separate isotropic from anisotropic diffusional variance [13]. This enables the separation of diffusional variance, microscopic anisotropy, and orientation coherence, which cannot be achieved by conventional diffusion encoding [12, 13, 14].

However, diffusion in biological tissue is rarely Gaussian [15, 16], warranting experiments to be described by quantities beyond the b‐tensor to enable a more complete description of the signal. Such effects are commonly referred to as diffusion‐time effects and have been used to produce several imaging biomarkers [16, 17, 18]. Naturally, if such effects are present but ignored, they may lead to inaccurate quantification and interpretation of diffusion parameters in terms of microstructure features. In conventional dMRI measurement, where single diffusion encoding is applied along one direction per shot, the waveform shape and timing are usually kept constant, and only the gradient amplitude and encoding direction are varied [19]. As a result, diffusion‐time effects are present but uniform across measurements, introducing a constant offset rather than a variable confound. They become an additional encoding dimension, whether intended or not, only when waveform shape or timing is varied across measurements.

State‐of‐the‐art tensor‐valued diffusion encoding uses numerically optimized waveforms (NOW) to maximize encoding efficiency [20, 21]. Although NOW minimizes the encoding duration for the target b‐value and facilitates practical implementation across a wide range of MRI systems [22], it does not account for spectral anisotropy (variable restriction weighting across projections of a single waveform) or spectral mismatch (different restriction weighting across different waveforms) [23]. Thus, time‐dependent diffusion may confound the measurement and downstream quantification [24]. In practice, spectral anisotropy leads to rotational variance despite a spherical b‐tensor [25, 26], and spectral mismatch causes different apparent diffusivities measured by different waveforms [23, 24, 27], both of which violate the assumptions of representations that depend only on the b‐tensor.

Recently, Lasič et al. (2025) introduced the spectral principal axis system (SPAS) to address spectral matching by tuning the restriction weighting of an LTE to an STE in the spectral domain [23]. However, Hamilton et al. (2026) showed that the practical outcome can still depend on how spectral tuning is implemented, since different matching strategies led to slightly different quantitative diffusion measures [28]. This suggests that matching STE and LTE under time‐dependent diffusion is not only a question of ‘tuning’ in principle, but also of how the matched pair is constructed in practice. Thus, there is a need for novel and straightforward ways to optimize sets of waveforms that improve spectral anisotropy and matching under conditions of time‐dependent diffusion.

We propose a novel gradient waveform design and optimization strategy called double isotropic matched encoding (DIME) that aims to eliminate spectral anisotropy and mismatch to achieve rotation‐invariant STE and a spectrally matched LTE. The framework also accounts for imbalanced concomitant gradients as well as peripheral and cardiac nerve stimulation.

## 2 Theory

### 2.1 Diffusion and Restriction Weighting: Spectral Origins of b and m

Diffusion encoding in one dimension can be described by the random phase accumulated by spins in a time‐varying magnetic field gradient g(t). Under the Gaussian approximation, the attenuation of signal S in the frequency domain takes a form that separates waveform‐dependent encoding from tissue‐dependent diffusion dynamics [29]

(1) $$\ln\frac{S}{S_{0}}=-\frac{1}{2\pi }\int_{-\infty }^{\infty }{\left\|Q(\omega )\right\|}^{2}D(\omega )\;d\omega .$$

Here $S_{0}$ is the baseline signal, D(ω) is the diffusion spectrum and Q(ω) is the encoding spectrum, that is, the Fourier transform of the dephasing vector

(2) $$q(t)=\gamma \int_{0}^{t}g(t^{\prime })\;dt^{\prime }.$$

The signal attenuation is determined by the spectral overlap between the encoding power spectrum ${\left\|Q(\omega )\right\|}^{2}$ and the diffusion spectrum D(ω), which describes how the apparent diffusivity of the tissue varies with encoding frequency.

Time‐dependent effects arise when the diffusion spectrum varies with ω, as seen in Figure 1. When D(ω) is approximately constant over the interval where the encoding power is nonnegligible, the signal is determined by the diffusion weighting strength, or b‐value. By contrast, if D(ω) varies, the waveform timing and shape of the encoding power spectrum $||Q(\omega )|{|}^{2}$ becomes important. For example, two waveforms with the same b‐value may yield different attenuation because they weight different parts of the diffusion spectrum (Figure 2).

**FIGURE 1:**
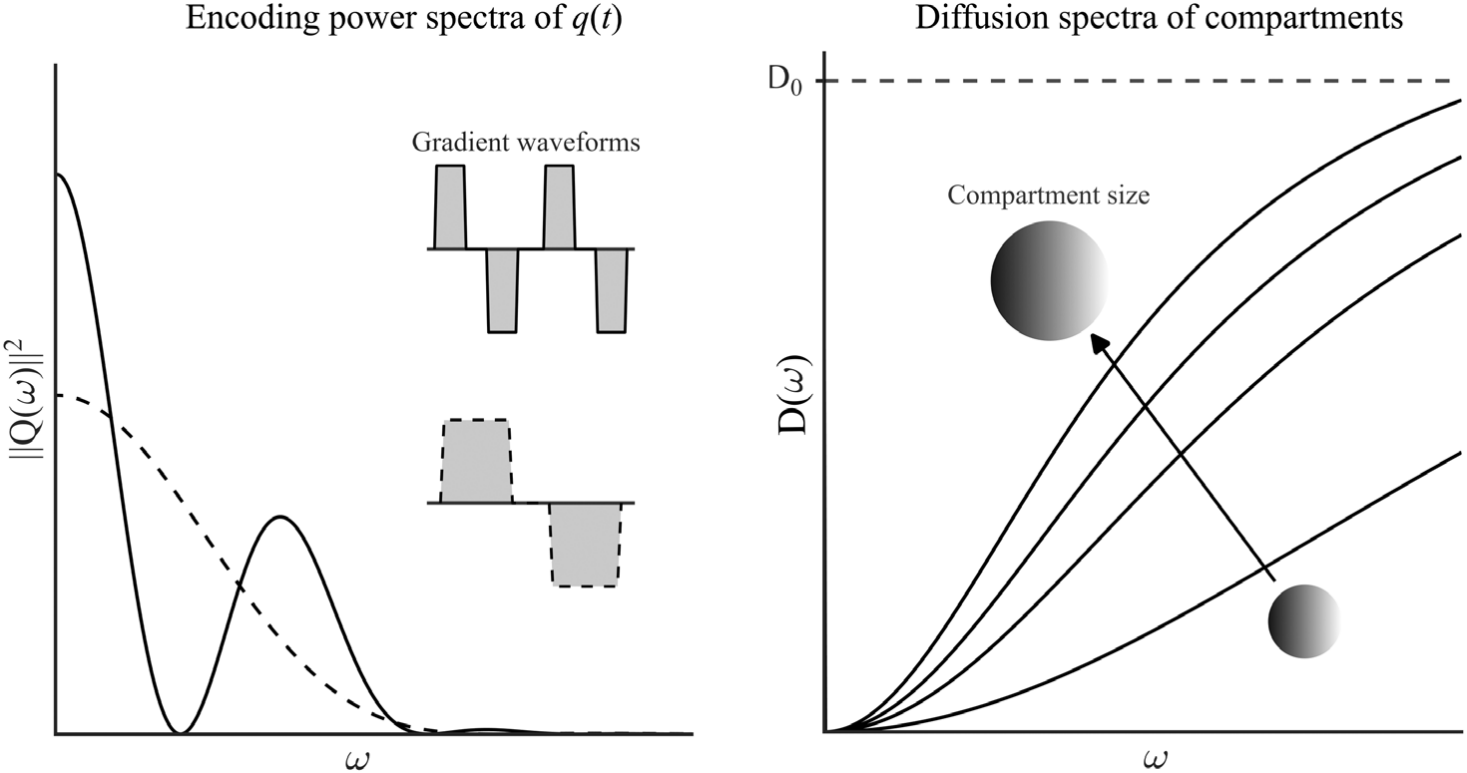
Diffusion attenuation is determined by the spectral overlap between the encoding power spectrum $||Q(\omega )|{|}^{2}$ and the compartment diffusion spectrum D(ω). The encoding power spectrum describes how a given gradient waveform distributes diffusion weighting across frequency, and thus across effective diffusion time scales. The diffusion spectrum describes how the apparent diffusivity of the compartment varies with frequency and size. For large compartments, D(ω) is nearly uniform and approaches the bulk diffusivity $D_{0}$ over the encoding bandwidth. In smaller compartments, boundary interactions suppress D(ω) at low frequencies, corresponding to the reduced apparent diffusivity at long diffusion times. Smaller compartments therefore exhibit stronger low‐frequency suppression than larger ones.

**FIGURE 2:**
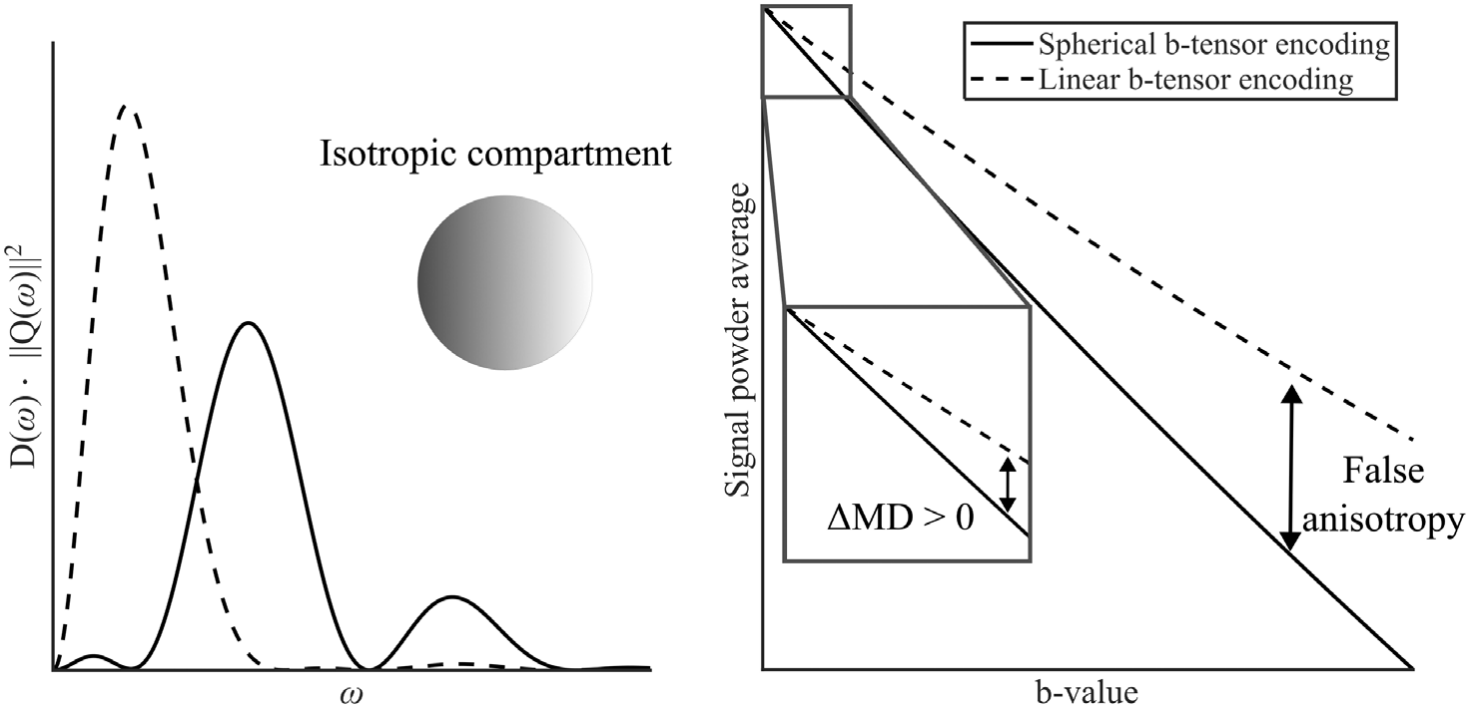
Consequence of time‐dependent diffusion when comparing different b‐tensor shapes. When diffusion is Gaussian, powder‐averaged LTE and STE signals that are matched in diffusion weighting (b‐value) probe the same MD and therefore coincide; any remaining difference can be attributed to anisotropic diffusion. When diffusion is restricted, however, the diffusion spectrum D(ω) becomes frequency‐dependent. At the same time, LTE and STE can have different encoding power spectra $||Q(\omega )|{|}^{2}$. Consequently, they weight the same diffusion spectrum differently, which changes the signal and apparent diffusivity. Furthermore, their signal versus b‐value can diverge, as expected in the presence of diffusion anisotropy, despite containing only isotropic restrictions. This is a confounding effect, for example when estimating microscopic diffusion anisotropy (μFA) [23, 24, 27].

At low frequencies, where molecular displacements have sampled the structural correlation length of the medium, the diffusion spectrum follows a power law [30, 31, 32]

(3) $$D(\omega )\approx D_{\infty }+C_{p}|\omega {|}^{p},$$

where $C_{p}$ is a sample‐dependent coefficient, $D_{\infty }$ the long‐time (low‐frequency) apparent diffusion coefficient, and the frequency exponent p is related to the structural class of the medium [30]. Closed and fully restricted compartments produce p=2 with $D_{\infty }=0$ [32], while hindered diffusion in disordered media produces smaller exponents [30, 31]. Inserting Equation (3) into Equation (1) separates the attenuation into a sample‐dependent coefficient and a waveform‐dependent spectral moment

(4) $$\ln\frac{S}{S_{0}}=-\left(bD_{\infty }+m_{p}C_{p}\right),$$

where m is the spectral moment of order p of the encoding power spectrum $||Q(\omega )|{|}^{2}$, according to

(5) $$m_{p}=\frac{1}{2\pi }\int_{-\infty }^{\infty }|\omega {|}^{p}||Q(\omega )|{|}^{2}\;d\omega .$$

The diffusion weighting factor, or b‐value, is the zeroth moment of the encoding power spectrum

(6) $$b=m_{0}=\frac{1}{2\pi }\int_{-\infty }^{\infty }{\left\|Q(\omega )\right\|}^{2}\;d\omega =\int_{0}^{\tau }q^{2}(t)\;dt,$$

and, likewise, the restriction weighting factor, or m‐value, is its second moment [32, 33]

(7) $$m\equiv m_{2}=\frac{1}{2\pi }\int_{-\infty }^{\infty }\omega^{2}{\left\|Q(\omega )\right\|}^{2}\;d\omega =\gamma^{2}\int_{0}^{\tau }g^{2}(t)\;dt,$$

where τ is the total encoding duration. Equation (7) follows from Parseval's theorem, and the case where p=2 can be written in terms of the effective gradient waveform and corresponds to fully restricted diffusion in closed compartments, for which the restriction contribution to the signal depends on the waveform only through the m‐value. As the encoding power shifts to higher frequencies, the encoding spectrum becomes wider and m/b becomes larger.

The spectral representation shows how the waveform shape determines the total diffusion weighting b and the restriction weighting m. To describe these quantities for general gradient waveforms in three dimensions, we use the vector‐valued gradient waveform g(t) and the corresponding dephasing vector q(t) [9], from which the b‐tensor can be defined as

(8) $$B=\int_{0}^{\tau }q(t)⊗q(t)\;dt,$$

where ‘⊗’ denotes the outer product. Similarly, the restriction weighting m‐tensor is defined as [33]

(9) $$M=\gamma^{2}\int_{0}^{\tau }g(t)⊗g(t)\;dt.$$

Using the m‐tensors as an example, we note that the size of a tensor is given by its trace

(10) $$m=\underset{i}{\sum }M_{ii},$$

and the shape, or anisotropy, is related to the normalized variance of the eigenvalues [27, 33]

(11) $$\zeta_{m}=\frac{1}{2m^{2}}\underset{ij}{\sum }\left(3M_{ij}^{2}-M_{ii}M_{jj}\right).$$

The shape of M quantifies the spectral anisotropy of low‐frequency restriction weighting, and its eigenvectors define the principal axes of the second‐order spectral moment tensor (see Equation (7)), analogous to the SPAS framework of Lasič et al. [23].

Following this formulation, we describe the diffusion‐weighted signal within a voxel using the first‐order macroscopic representation [27, 33]

(12) $$\ln\frac{S}{S_{0}}\approx -B:\left\langle D_{\mu }\right\rangle -M:\left\langle R_{\mu }\right\rangle ,$$

where ‘:’ denotes the double inner product, ‘⟨·⟩’ the average operator over microdomains within the voxel, and $D_{\mu }$ and $R_{\mu }$ are the long‐time diffusion‐ and restriction effect tensors of microdomains denoted with a subscript μ.

Isotropic diffusion encoding therefore requires that both B and M in Equation (12) are isotropic, or spherical, such that

(13) $$B=\frac{b}{3}\;I,\;M=\frac{m}{3}\;I,$$

where I is the identity matrix. Furthermore, equal restriction weighting, whereby different waveforms report the same mean diffusivity $\text{MD}=\frac{1}{3}\sum_{i}D_{ii}$ (where D is the diffusion tensor), requires that waveforms have the same trace of the m‐tensor for a given b‐value, that is, the waveforms have equal

(14) $$\frac{m}{b}=\frac{\sum_{i}M_{ii}}{\sum_{i}B_{ii}}.$$

Under these conditions, Equation (12) yields a signal that is rotation‐invariant within the low‐frequency approximation and reflects the same MD across waveforms.

### 2.2 Gradient Waveform Balance and Concomitant Dephasing

In a spin‐echo experiment, the refocusing pulse inverts the sign of the accumulated phase, and accurate diffusion encoding requires that the dephasing of stationary spins vanishes at the echo time. This is called the spin‐echo condition [34], and imposes that all waveforms are balanced according to

(15) $$k=\int_{0}^{\tau }h(t)\;g(t)\;dt=0,$$

where k is the dephasing vector and h(t) is a sign function taking on the values ±1. However, due to the laws described by Maxwell's equations, the desired gradient waveform $g_{d}(t)$, is accompanied by position‐dependent concomitant gradients $g_{c}(t,r)$ [21, 35], such that the actual gradient waveform becomes

(16) $$g(t,r)=g_{d}(t)+g_{c}(t,r),$$

where

(17) $$g_{c}(t,r)\approx \frac{1}{4B_{0}}\left[\begin{array}{lll} g_{z}^{2}(t) & 0 & -2\;g_{x}(t)g_{z}(t)\\ 0 & g_{z}^{2}(t) & -2\;g_{y}(t)g_{z}(t)\\ -\;2\;g_{x}(t)g_{z}(t) & -2\;g_{y}(t)g_{z}(t) & 4g_{x}^{2}(t)+4g_{y}^{2}(t) \end{array}\right]\cdot r.$$

Here, $g_{x}(t),\;g_{y}(t)$ and $g_{z}(t)$ are the components of the desired gradient waveform $g_{d}(t)$, and we assume the convention that the main magnetic field has amplitude $B_{0}$ and points along the z‐axis. If the desired gradient waveform is asymmetric, the concomitant gradients may violate Equation (15), causing signal dephasing (k≠0) and deleterious effects on parameter estimation [21, 36, 37]. Thus, gradient waveform balance requires that both the desired and concomitant gradient waveforms are balanced.

## 3 Methods

Here, we first describe the design of DIME waveforms as well as reference waveforms based on the NOW framework. To assess their performance in terms of spectral anisotropy and spectral matching, all waveforms were evaluated in simulations of simple geometries as well as histology‐derived substrates.

### 3.1 Design of the DIME Waveforms

DIME waveforms were designed to satisfy four requirements: (i) rotationally invariant STE under time‐dependent diffusion, (ii) matched restriction weighting across STE and LTE, (iii) gradient balance with concomitant‐gradient compensation, and (iv) compliance with cardiac and peripheral nerve stimulation limits.

Our initial STE waveform design uses three trapezoidal pulse pairs (Figure 3), inspired by waveform pattern I presented by Mori and van Zijl [38], that is, a triple diffusion encoding arrangement. The outer pairs are bipolar while the central pair is monopolar with one trapezoid on either side of the refocusing pulse. Additionally, the central pair is separated by a pause time $t_{p}$ to accommodate the refocusing pulse, and each trapezoid pair is constructed such that the two trapezoids within it are time‐reversals of each other. We note that the three pulse pairs are arranged to be nonoverlapping in time with regard to both g(t) and q(t), which means that B and M have no off‐diagonal elements and can be calculated independently (Supporting Information, Section B).

**FIGURE 3:**
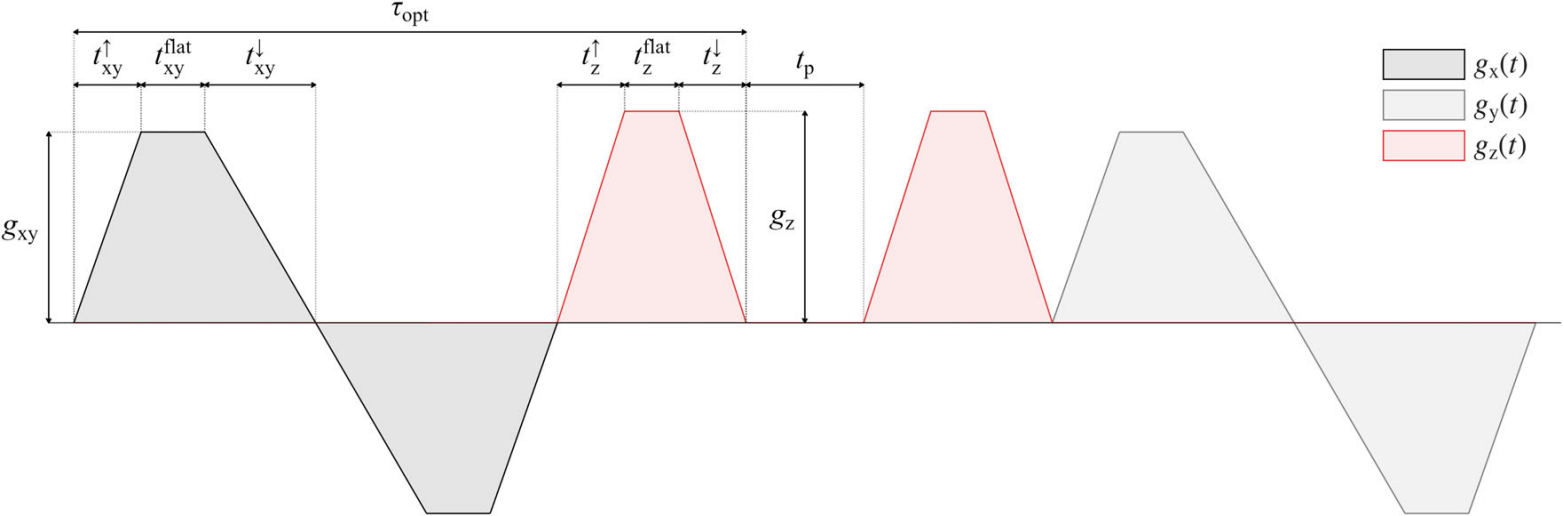
The proposed gradient waveform design and its parameters. The encoding comprises three nonoverlapping trapezoidal pulse pairs: two identical outer bipolar pairs applied along orthogonal in‐plane axes (x and y) within the plane transverse to the main magnetic field ($B_{0}$), each with amplitude $g_{xy}$, ramp times $t_{xy}^{↑}$ and $t_{xy}^{↓}$, and flat time $t_{xy}^{\text{flat}}$; and one central pair parallel to $B_{0}$, with amplitude $g_{z}$, ramp times $t_{z}^{↑}$ and $t_{z}^{↓}$, and flat time $t_{z}^{\text{flat}}$, separated by a pause time $t_{p}$ that accommodates the refocusing pulse. Each encoding block before and after the refocusing pulse is confined to an encoding duration $\tau_{\text{opt}}$ used during optimization. The flat‐top durations $t_{xy}^{\text{flat}}$ and $t_{z}^{\text{flat}}$ are jointly determined by $\tau_{\text{opt}}$ and the ramp times, with the remaining time partitioned between the xy‐ and z‐trapezoids. In each outer pair, the second trapezoid is the sign‐inverted and time‐reversed counterpart of the first. By contrast, the two central trapezoids have the same polarity and are related by time reversal about the refocusing pulse. The outer pairs are identical apart from direction, reducing the waveform to a low‐dimensional parametrization for optimization.

#### 3.1.1 Double Rotational Invariance and Matched Restriction Weighting

We seek a gradient waveform with isotropic encoding for Gaussian and restricted diffusion, that is, one that simultaneously produces a spherical b‐tensor as well as a spherical m‐tensor Equation (13). The shape of these tensors is controlled by the temporal trajectory of the waveform. In the proposed design, the nonoverlapping waveform components and their orthogonal directions make both B and M diagonal, so rotational invariance, that is, $\zeta_{b}\approx 0$ and $\zeta_{m}\approx 0$, reduces to matching their diagonal elements across axes, such that $b_{x}=b_{y}\approx b_{z}$ and $m_{x}=m_{y}\approx m_{z}$.

To yield a pair of STE and LTE waveforms that are matched with respect to their restriction weighting, they must have equal m/b Equation (14). We constructed the LTE waveform from the STE waveform by projecting it onto a vector u, according to

(18) $$g_{\text{LTE}}(t)=g_{\text{STE}}(t)\cdot u.$$

Because the three encoding blocks do not overlap in time, B and M are both diagonal, and isotropy makes them proportional to the identity, with $B_{ii}=b/3$ and $M_{ii}=m/3$. The projection in Equation (18) then yields

(19) $$b_{\text{LTE}}=\underset{i}{\sum }u_{i}^{2}B_{ii}=\frac{b}{3}||u|{|}^{2},\;m_{\text{LTE}}=\underset{i}{\sum }u_{i}^{2}M_{ii}=\frac{m}{3}||u|{|}^{2}.$$

Two properties follow directly. First, $m_{\text{LTE}}/b_{\text{LTE}}=m/b$ for any vector u, so every projection preserves the restriction weighting. Second, $b_{\text{LTE}}$ depends on the projection only through $||u|{|}^{2}$, and equals b when $||u|{|}^{2}=3$. The choice u=(±1,±1,±1) meets this condition by weighting the three orthogonal blocks equally, so that each block appears in the LTE at the same gradient amplitude as in the STE. The LTE therefore attains the same b‐value as the STE over the same encoding duration and at the same peak amplitude, and so inherits its encoding efficiency. The sign combinations of u are equivalent in b and m, but differ in the shape of q(t) and therefore the encoding power spectrum. We therefore selected a sign combination that makes the two outer pairs antiparallel (u=(−1,1,1)), which suppresses their interference with the central pair and yields the LTE spectrum closest to that of the STE.

We also propose that this method can be generalized to produce a matched LTE from any waveform with arbitrary M. The projection u that yields a matching m/b can be found by eigenvalue decomposition of M, after which the gradient waveform from each principal axis is summed in equal weights. This is equivalent to Equation (18) where $g_{\text{STE}}(t)$ has first been rotated such that its m‐tensor is in the principal axis system. Note that, unlike DIME, this projection does not generally maintain the encoding efficiency and may therefore lead to an LTE waveform that cannot reach the same range of b‐values as the corresponding STE waveform given the same encoding time.

#### 3.1.2 Waveform Balance With Concomitant Gradient Compensation

Since DIME waveforms are asymmetric around the refocusing pulse, it is imperative to ensure that balance and concomitant gradients are controlled [21, 35]. As can be seen in Figure 3, the desired gradient waveform is balanced since each pair of pulses is matched and has $k_{d}=0$ Equation (15). Thereby, the desired gradient waveform always fulfills the spin‐echo condition.

The waveform design also enables balancing the concomitant gradient, such that $k_{c}=0$ Equation (15). As can be seen from Equation (17), certain symmetries enable cancellation despite the asymmetry. Following Szczepankiewicz et al. [39], we constrain the two outer pulse pairs to have the same shape and amplitude and lie in the plane orthogonal to the main magnetic field (xy‐plane), while the central pulse pair is symmetric about the refocusing period and parallel to the main magnetic field (z‐direction). Thus, all contributions from Equation (17) vanish when integrated over time (Equation (15)). This follows from three properties: the symmetry of $g_{z}(t)$, the orthogonality $g_{x}(t)g_{z}(t)=g_{y}(t)g_{z}(t)=0$, and equal norms of $g_{x}^{2}(t)+g_{y}^{2}(t)$ before and after the refocusing pulse. This mode of compensating for concomitant gradient effects is called K‐nulling and is only fulfilled in a small set of rotations [39]. Thus, it is most compatible with gradient waveforms that do not need to be rotated, such as isotropic diffusion encoding [39, 40], for which a single fixed orientation already yields rotation‐invariant encoding.

The corresponding LTE waveform is also comprised by matching pulse pairs, and therefore also balanced with respect to the desired gradient waveform (Equation (15)). Moreover, using the optimal projection (Equation (18)) where u=(±1,±1,±1), it is also balanced with respect to the concomitant gradients for arbitrary rotations. This is because it follows the condition of ‘quadratic nulling’ [41], that is, it is symmetric with respect to $g^{2}(t)$.

#### 3.1.3 Compliance With Cardiac and Peripheral Nerve Stimulation Limits

To enable safe execution, the DIME waveforms are optimized to comply with nerve stimulation limits. Stimulation is mainly dependent on the time derivative of the gradient waveform, such that long periods of high slew rates are more likely to cause stimulation [42]. This means that gradient amplitudes and ramp times can be used to modulate stimulation levels. The trade‐off for not maximizing the slew rate is a lower encoding efficiency.

In this work, stimulation levels were predicted from the gradient waveform and scanner‐specific parameters using the SAFE model [43], as implemented in MATLAB [44]. Here, both peripheral and cardiac nerve stimulation $\left(n_{p}(t\right)$ and $n_{c}(t))$ were calculated as functions of time. SAFE was chosen for its computational efficiency and scanner specificity; however, we expect that implementations tailored to other vendors or models may be used in the optimization instead [45, 46]. We note that the stimulation level is only an indicator of the risk for stimulation, and acceptable waveforms are those whose norm never exceeds the specified safety limit.

#### 3.1.4 Numerical Optimization of DIME

We performed a numerical optimization of DIME waveforms given a fixed encoding duration before and after the refocusing pulse ($\tau_{\text{opt}}$), a maximum slew rate $\left(s_{\max}\right)$, a maximum gradient amplitude $\left(g_{\max}\right)$, and one pause time ($t_{p}$). The optimization has seven free parameters: two gradient amplitudes ($g_{xy}$, $g_{z}$), four ramp times ($t_{xy}^{↑}$, $t_{xy}^{↓}$, $t_{z}^{↑}$, $t_{z}^{↓}$), and one flat‐time‐partitioning parameter (β∈[0,1]). The flat‐top durations $t_{xy}^{\text{flat}}$ and $t_{z}^{\text{flat}}$ are jointly determined by $\tau_{\text{opt}}$ and the ramp times, with the remaining time partitioned between the xy‐ and z pairs by β.

The optimization was implemented in fmincon, MATLAB (The MathWorks Inc., Natick, MA, USA, version R2025b) using the sequential quadratic programming algorithm. Constraints on b‐ and m‐tensor isotropy were evaluated analytically from the waveform parameters, while nerve stimulation constraints were evaluated numerically after waveform interpolation using the SAFE model [43, 44]. To avoid local minima, we used a multi‐start scheme with an initial guess selected randomly near a heuristic set of parameters. The objective was to maximize the attainable b‐value subject to the following constraints:

i. gradient amplitude, $g_{xy},\;g_{z}<g_{\max}$;
ii. slew rate, $s_{xy},\;s_{z}<s_{\max}$;
iii. isotropic b‐tensor, $\sqrt{\zeta_{b}}<0.01/3$;
iv. isotropic m‐tensor, $\sqrt{\zeta_{m}}<0.01/3$;
v. stimulation limits, $|n_{p}(t)|,|n_{c}(t)|<95\%$.

The matching of restriction weighting, waveform balance, and concomitant gradient compensation are guaranteed by the underlying waveform design and are not part of the optimization.

Stimulation constraints were evaluated numerically with the SAFE model after rasterizing waveforms to a resolution of 46 μs. For STE waveforms, nerve stimulation was assessed using the Euclidean norm of the predicted PNS and CNS levels, $n_{p}(t)$ and $n_{c}(t)$. This is sufficient because the STE waveform is not intended to rotate. By contrast, stimulation levels from the LTE waveform were evaluated along the three physical gradient axes independently. This ensures that the resulting waveform will comply with the stimulation threshold regardless of rotation. Where possible, both peripheral and cardiac stimulation were considered, and their values were required to be below 95% of the safety limit. Notably, this threshold can be set to any value to comply with various tolerances and safety limits.

### 3.2 Design of the NOW Waveforms

As a benchmarking reference, three STE and LTE waveform pairs were generated using the NOW toolbox [20], with Maxwell compensation [21]. In each pair, the STE is optimized to maximize the b‐value for a given encoding duration under gradient amplitude and slew‐rate constraints, and the LTE is obtained by projecting the STE onto a chosen direction. The three sets differ with respect to encoding duration and the projection used to construct the LTE, according to:

i. **NOW‐OE** (optimal efficiency): the STE uses the NOW‐optimal (shorter) encoding duration, and the LTE projection is chosen to maximize the b‐value of the LTE.
ii. **NOW‐RM** (restriction matched): the same STE as NOW‐OE, with the LTE projection instead chosen to match m/b according to Equation (18).
iii. **NOW‐ET** (encoding‐time matched): the STE encoding duration is extended to match that of the corresponding DIME waveform, with the LTE projection chosen to maximize the b‐value, as in NOW‐OE.

NOW‐OE represents the state‐of‐the‐art implementation, appropriate when restriction effects can be ignored; NOW‐RM is the best restriction‐matched pair attainable from a NOW waveform; and NOW‐ET matches the encoding duration of DIME. Since a longer encoding duration lowers m/b and thereby reduces restriction sensitivity, NOW‐ET isolates the effect of duration from that of the enforced isotropy and matching that distinguish DIME. Each waveform was interpolated to a temporal resolution of 46 μs.

### 3.3 Evaluation of DIME by Simulations in Ideal and Realistic Substrates

#### 3.3.1 Waveform and Encoding Efficiency

Eight waveform pairs were optimized for two gradient systems with maximal gradient amplitudes of $g_{\max}=80$ and 200mT/m, maximal slew rates of 200 T/m/s, with encoding durations set to achieve maximum b‐values of 2 ms/$\mu m^{2}$. For DIME, the fixed optimization parameters were $\tau_{\text{opt}}=28\;\text{ms}$ and $t_{p}=7\;\text{ms}$ on the 200 mT/m system, and $\tau_{\text{opt}}=38\;\text{ms}$ and $t_{p}=8\;\text{ms}$ on the 80 mT/m system. SAFE model stimulation parameters were based on corresponding MRI systems from Siemens Healthineers (MAGNETOM Prisma and Cima.X, Siemens Healthineers, Forchheim, Germany). Both systems have specifications for PNS, but only the 200 mT/m system has parameters for CNS prediction. Thus, the optimization of waveforms at 80 mT/m only considered PNS. Gradient amplitudes were scaled to yield $b\in [0,\;0.1,\;0.5,\;1,\;2]\;\text{ms}/\mu m^{2}$.

Encoding efficiency under the prescribed hardware constraints was quantified using the dimensionless metric [39, 47]

(20) $$\kappa =\frac{4b}{\gamma^{2}g_{\max}^{2}\tau^{3}}.$$

Here, larger κ indicates more efficient diffusion weighting for a given maximum gradient amplitude $g_{\max}$ and diffusion‐encoding duration τ. Note that κ is invariant under uniform rescaling of the time axis and therefore characterizes a waveform shape rather than duration.

The total waveform durations $\tau =2\tau_{\text{opt}}+t_{p}$, restriction‐weighting ratios m/b, m‐tensor shapes $\zeta_{m}$, and encoding efficiencies κ are summarized in Table 1.

**TABLE 1:** Waveform properties for the four encoding pairs evaluated on each of the two gradient systems. The duration τ is the diffusion‐encoding duration for the full waveform (including the pause time $t_{p}$), and κ quantifies the waveform efficiency. The restriction‐weighting ratio m/b is given in $10^{4}\;s^{-2}$. The restriction tensor shape $\zeta_{m}$ refers to the STE waveform. DIME is the only waveform with an isotropic m‐tensor ($\zeta_{m}=0$), whereas the reference NOW‐based STE waveforms exhibit high spectral anisotropy. As intended, both DIME and NOW‐RM are matched in terms of m/b.

$g_{\max}$ [mT/m]	Waveform	τ [ms]	m/b [10 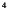 s 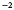 ]		$\sqrt{\zeta_{m,\text{STE}}}$	κ·100 [%]	
	STE	LTE	STE	LTE
200	DIME	63	2.84	2.84	0.003	1.19	1.19
	NOW‐OE	42	4.56	0.75	0.443	3.19	9.22
	NOW‐RM	42	4.56	4.56	0.443	3.19	1.18
	NOW‐ET	63	2.38	0.37	0.446	4.27	6.24
80	DIME	84	1.35	1.35	0.003	2.95	2.95
	NOW‐OE	71	1.31	0.23	0.439	4.82	10.9
	NOW‐RM	71	1.31	1.31	0.439	4.82	1.84
	NOW‐ET	84	0.90	0.15	0.442	5.21	7.93

To supply another, more tangible, parameter of encoding efficiency, κ was also converted to the total encoding duration required to reach $b=2\;\text{ms}/\mu m^{2}$ at system $g_{\max}$, according to

(21) $$\tau_{\text{enc}}={\left(\frac{4b}{\gamma^{2}g_{\max}^{2}\kappa }\right)}^{1/3},$$

with shorter $\tau_{\text{enc}}$ indicating higher efficiency with a potential opportunity for reduced echo times.

#### 3.3.2 Analysis of Rotation Invariance and Waveform Matching in Cylinders and Spheres

To simulate the effect of sample rotations, each optimized waveform was rotated by a common set of 100 rotation matrices generated by geometric filter optimization (GFO) as described by Jespersen and Szczepankiewicz [48], available at: http://github.com/Neurophysics-CFIN/GFO. The waveform was rotated rather than the sample for convenience, as these are equivalent. This rotation scheme is used to ensure that all encoding features are well‐distributed in space. The b‐values were set by scaling the gradient waveform amplitude, preserving the timing and tensor shape. The DIME optimizer is available as open‐source software: http://github.com/filip-szczepankiewicz/fwf_sequence_tools.

To quantify spectral anisotropy and matching effects under controlled microstructure, signals were generated using analytical calculations following Lundell & Lasič [33] and Nilsson et al. [32] and evaluated using Equation (1) (Supporting Information, Section C), as well as numerical Monte Carlo simulations using the Disimpy framework [49]. Each Monte Carlo simulation launched $N_{p}=10^{6}$ walkers and advanced them for $N_{t}=2730$ time steps using a temporal resolution Δt=46μs and free diffusivity $D=2\;\mu m^{2}/\text{ms}$.

We examined infinitely long cylinders with radii R∈[0.1,90]μm and spheres with radii R∈[3,90]μm. For cylinders, Monte Carlo simulations were restricted to R≥3μm due to the increasing computational cost at smaller radii, with analytical calculations used to cover the full range from R=90 μm down to R=0.1 μm. For spheres, the smallest radii accessible by Monte Carlo were sufficient to cover the range of interest, and analytical calculations were not required. The provided code also supports analytical calculation of sphere signals should smaller radii be of interest; see data availability statement.

To assess spectral anisotropy through rotational invariance, each STE waveform was first scaled in amplitude to achieve $b=1\;\text{ms}/\mu m^{2}$ and then applied to cylindrical substrates (chosen as the worst‐case scenario for rotational variance due to their maximal diffusion anisotropy) in all 100 rotations. Rotational invariance was quantified as the coefficient of variation (CV) of the simulated signal across rotations.

To determine waveform spectral matching, signals were generated in spherical substrates across the full set of simulated b‐values and over the same set of 100 rotations. The MD was then estimated by fitting Equation (22) to the powder‐averaged signal such that

(22) $$S=S_{0}\exp\left(-bMD+\frac{1}{2}b^{2}V-\frac{1}{6}b^{3}\xi \right),$$

where $S_{0}$ is the baseline signal, and V and ξ represent diffusional variance and skewness, respectively [50], but were only included to absorb higher‐order terms to improve the accuracy of the estimation of MD. The degree of mismatch is quantified in terms of the MD difference, according to ΔMD = MD
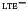
MD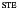.

#### 3.3.3 Analysis of Waveform Matching in Histology‐Derived Substrates

To complement the ideal substrates, we generated trajectories from realistic histology‐derived substrates using the Histo‐µSim framework by Grigoriou et al. [51]. Briefly, the software converts stained 2D micrographs into barrier maps and propagates random walkers between time‐indexed gradient samples, producing two‐dimensional trajectories that capture realistic cell sizes, packing densities, and extracellular spaces. These trajectories were used for every encoding so that differences in the fitted metrics arise solely from waveform design. Two intracellular diffusivities $D_{\text{IC}}$ were examined on 18 substrates spanning diverse tissue morphologies, ranging from noncancerous liver tissue to heterogeneous cancers including fibrosis and necrosis. Simulations were performed with $D_{\text{IC}}=0.8\;\mu m^{2}/\text{ms}$ and $1.9\;\mu m^{2}/\text{ms}$, with corresponding extracellular diffusivities $D_{\text{EC}}=1.9\;\mu m^{2}/\text{ms}$ and $2.45\;\mu m^{2}/\text{ms}$. Simulations were run for all substrates and diffusivity combinations, yielding 36 unique tissue‐like environments in which MD matching between STE and LTE was evaluated for each waveform pair.

Each gradient waveform g(t) was rotated by each of the 100 GFO rotation matrices, projected onto the xy‐plane by discarding the z‐component, and rescaled in amplitude to maintain the target b‐value. For each spin trajectory $r_{n}(t)$ within the substrate and the resulting 2D gradient waveform, the accumulated phase $\varphi_{n}$ at the end of the encoding was estimated as

(23) $$\varphi_{n}=\gamma \overset{N_{t}}{\underset{k=1}{\sum }}g(t_{k})r_{n}(t_{k})\Delta t.$$

The diffusion‐weighted signal S was obtained as the magnitude of the ensemble‐averaged final spin state

(24) $$S=\left|\frac{1}{N_{p}}\overset{N_{p}}{\underset{n=1}{\sum }}e^{i\varphi_{n}}\right|.$$

For each substrate type, MD was estimated by fitting Equation (22) to S and the degree of waveform matching was quantified by ΔMD.

## 4 Results

The DIME optimization produced waveforms that satisfied the isotropy and matching constraints, yielding an STE with M=mI/3 and an LTE matched to the STE in m/b (see Table 1, Figure 4 and Figure S1 in Supporting Information). By contrast, the NOW‐based designs showed greater spectral anisotropy from an anisotropic M and larger spectral mismatch, particularly for the efficiency‐optimized NOW‐OE and encoding‐time‐matched NOW‐ET. All DIME waveforms satisfied the nerve stimulation safety limits as seen in Figure 5. At 200 mT/m, the waveform was largely restricted by CNS, with the LTE, STE norm, and STE z‐component approaching 95%, while at 80 mT/m the largest responses were PNS in the STE y‐, z‐, and norm.

**FIGURE 4:**
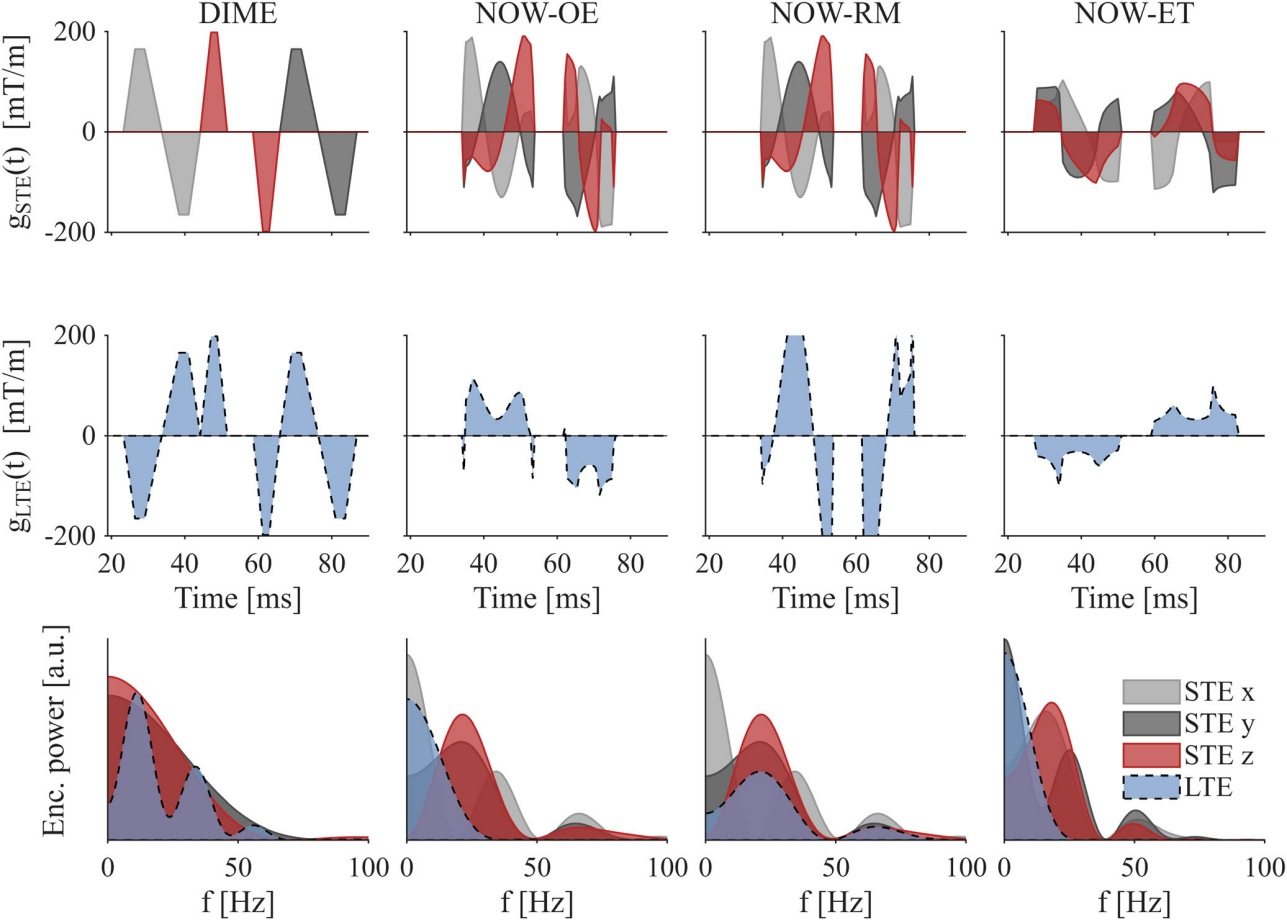
Optimized STE and LTE waveforms for the 200 mT/m system and their encoding power spectra, where STE x, y, and z refer to the gradient axes of the STE. DIME yields spectra with similar envelopes across STE projections and LTE, consistent with matched restriction sensitivity. By contrast, the NOW waveforms display marked spectral anisotropy between STE axes and stronger mismatch between STE and LTE spectra, indicating less uniform and less closely matched restriction sensitivity.

**FIGURE 5:**
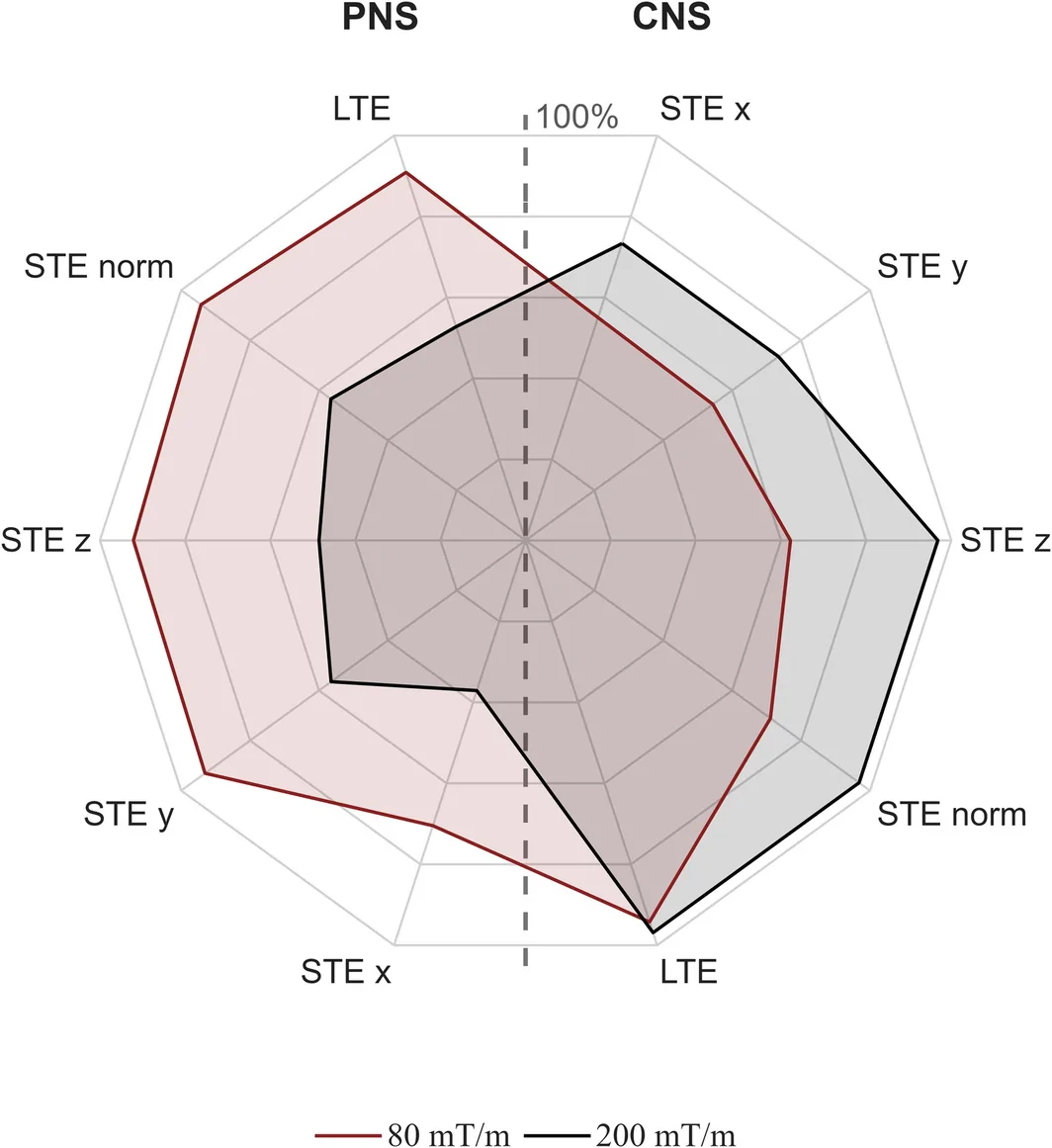
Predicted peripheral and cardiac nerve stimulation (PNS and CNS) levels for the DIME STE components (x,y,z and norm) and the matched LTE for 80 and 200 mT/m systems. All waveforms remained within the prescribed limit. On the 200 mT/m system, the main limiting factor was CNS, with the LTE, STE norm, and STE z‐component approaching 95% of the boundary. On the 80 mT/m system, only the PNS was constrained. The corresponding CNS levels were calculated based on the 200 mT/m‐system and may therefore be inaccurate.

DIME had the lowest rotational variance in cylinders across all waveform designs and gradient amplitudes (Figure 6). On the 80 mT/m system, the median (IQR) coefficient of variation was 2.0% (1.5%–3.3%) for DIME, 2.5% (1.8%–4.1%) for efficiency‐optimized/restriction‐matched NOW‐OE/NOW‐RM, and 2.8% (2.0%–4.4%) for encoding‐time‐matched NOW‐ET. On the 200 mT/m system, the corresponding values were 1.7% (1.2%–2.7%) for DIME, 2.0% (1.4%–3.5%) for NOW‐OE/NOW‐RM, and 2.3% (1.7%–4.0%) for NOW‐ET. This ordering was preserved across all waveform designs, gradient amplitudes, and cylinder radii. The dependence on the radius was nonmonotonic, with low CV at small radii, a maximum at approximately 10 to 15 μm, and a gradual decrease toward larger radii. In the small‐radius regime, the CV was nearly the same for all waveform pairs up to about 2 μm. For larger radii, the CV for NOW waveforms increased more rapidly, whereas DIME remained relatively low and stable up to approximately 4 μm, after which it also increased but reached a lower peak.

**FIGURE 6:**
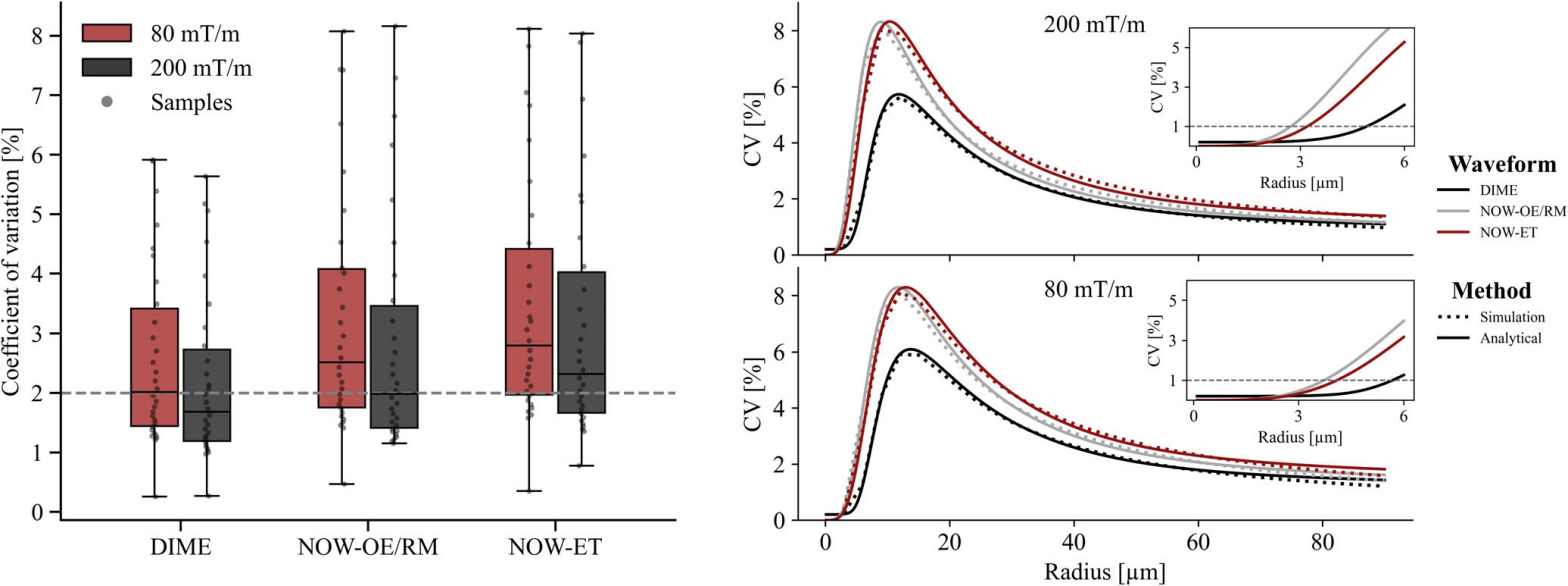
Rotational variance of the STE signal in a cylinder with variable radius, quantified by the coefficient of variation (CV) across 100 waveform rotations and $b=1\;\text{ms}/\mu m^{2}$. DIME yields the lowest rotational variance. Boxplots show CV distributions across radii for each waveform type and maximal gradient amplitude; lower CV indicates lower rotational variance. The right plots show CV as a function of cylinder radius. Although DIME consistently has the lowest rotational variance, all waveforms exhibit a dependence on cylinder radius. Solid and dashed curves are analytical and Monte Carlo–based results, respectively. NOW‐OE and NOW‐RM are identical because they have the same STE waveform. The insets highlight the small‐radius regime, where all waveforms exhibit similar rotational variance, together with a reference line at 1% CV.

DIME produced the smallest spectral mismatch between STE and LTE on both the 80 mT/m and 200 mT/m systems (Figure 7). Across radii, DIME produced closely matched MD values across STE and LTE, with a maximum absolute ΔMD of 0.08 μm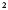/ms at R=21 μm for the 80 mT/m system and 0.07 μm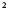/ms at R=18 μm for the 200 mT/m system. NOW‐RM also reduced the mismatch relative to the other NOW‐based designs, but ΔMD was larger, with maximal values of 0.13 μm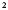/ms at R=15 μm for the 80 mT/m system and 0.09 μm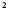/ms at R=15 μm for the 200 mT/m system. By contrast, NOW‐OE and NOW‐ET showed substantially larger differences between LTE and STE, each reaching a maximum absolute difference of 0.52 μm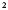/ms. For NOW‐OE, this maximum occurred at R=12 μm for the 80 mT/m system and R=9 μm for the 200 mT/m system, whereas for NOW‐ET it occurred at R=15 μm for the 80 mT/m system and R=12 μm for the 200 mT/m system. Thus, the same order of waveform performance as seen in cylinders was observed on both gradient systems in spheres: DIME showed the closest agreement between LTE and STE, NOW‐RM was intermediate, and NOW‐OE and NOW‐ET showed the largest mismatch.

**FIGURE 7:**
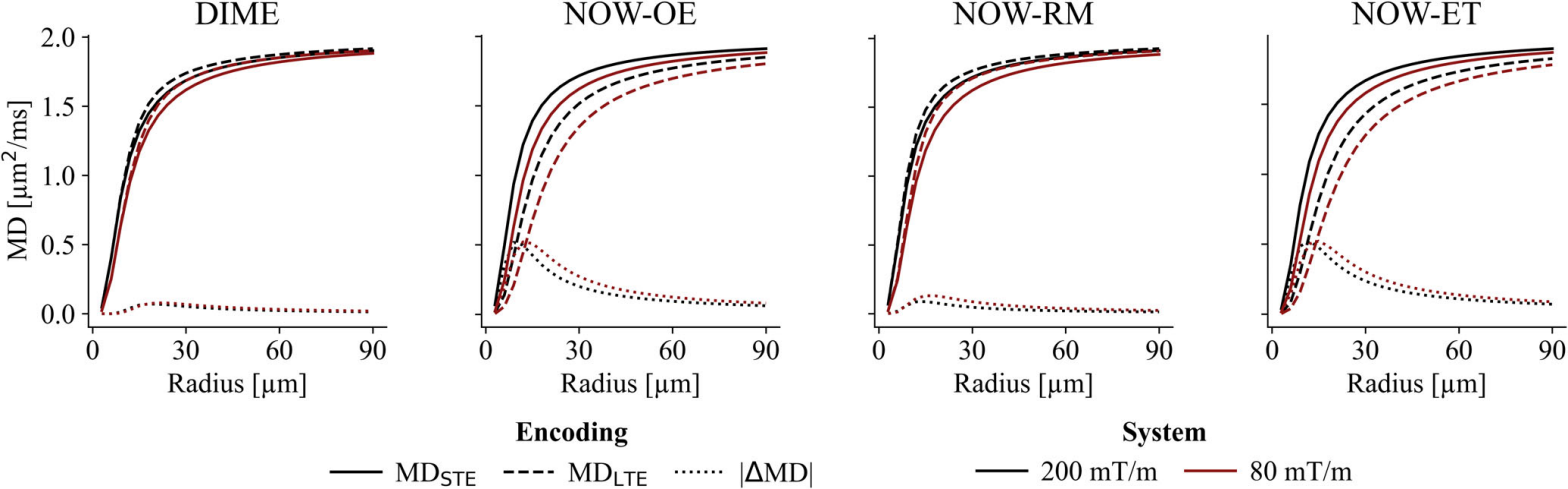
Mean diffusivity (MD) estimated from STE and LTE as a function of sphere radius R∈[3,90]μm, for the 80 mT/m (red) and 200 mT/m gradient systems. DIME yields the closest agreement between LTE‐ and STE‐derived MD across restriction sizes. NOW‐OE and NOW‐ET show a pronounced difference between LTE and STE, with a maximum mismatch around R≈10−20μm. The matched pair in NOW‐RM had a substantially lower mismatch relative to other NOW variants, but was less matched than DIME.

DIME also showed the closest agreement in MD between STE and LTE in histology‐derived substrates as seen in Figure 8. The mean difference was $\Delta \text{MD}=0.01\pm 0.01\;\mu m^{2}/\text{ms}$. Conversely, all NOW‐based waveforms showed larger ΔMD and broader distributions of residuals. NOW‐OE and NOW‐ET showed the largest mismatches, whereas NOW‐RM reduced the discrepancy relative to the other NOW‐based designs but remained less well matched than DIME. The corresponding distributions of residuals were $\Delta {\text{MD}}_{\text{NOW‐OE}}=-0.16\pm 0.10\;\mu m^{2}/\text{ms}$, $\Delta {\text{MD}}_{\text{NOW‐ET}}=-0.15\pm 0.10\;\mu m^{2}/\text{ms}$, and $\Delta {\text{MD}}_{\text{NOW‐RM}}=0.03\pm 0.02\;\mu m^{2}/\text{ms}$.

**FIGURE 8:**
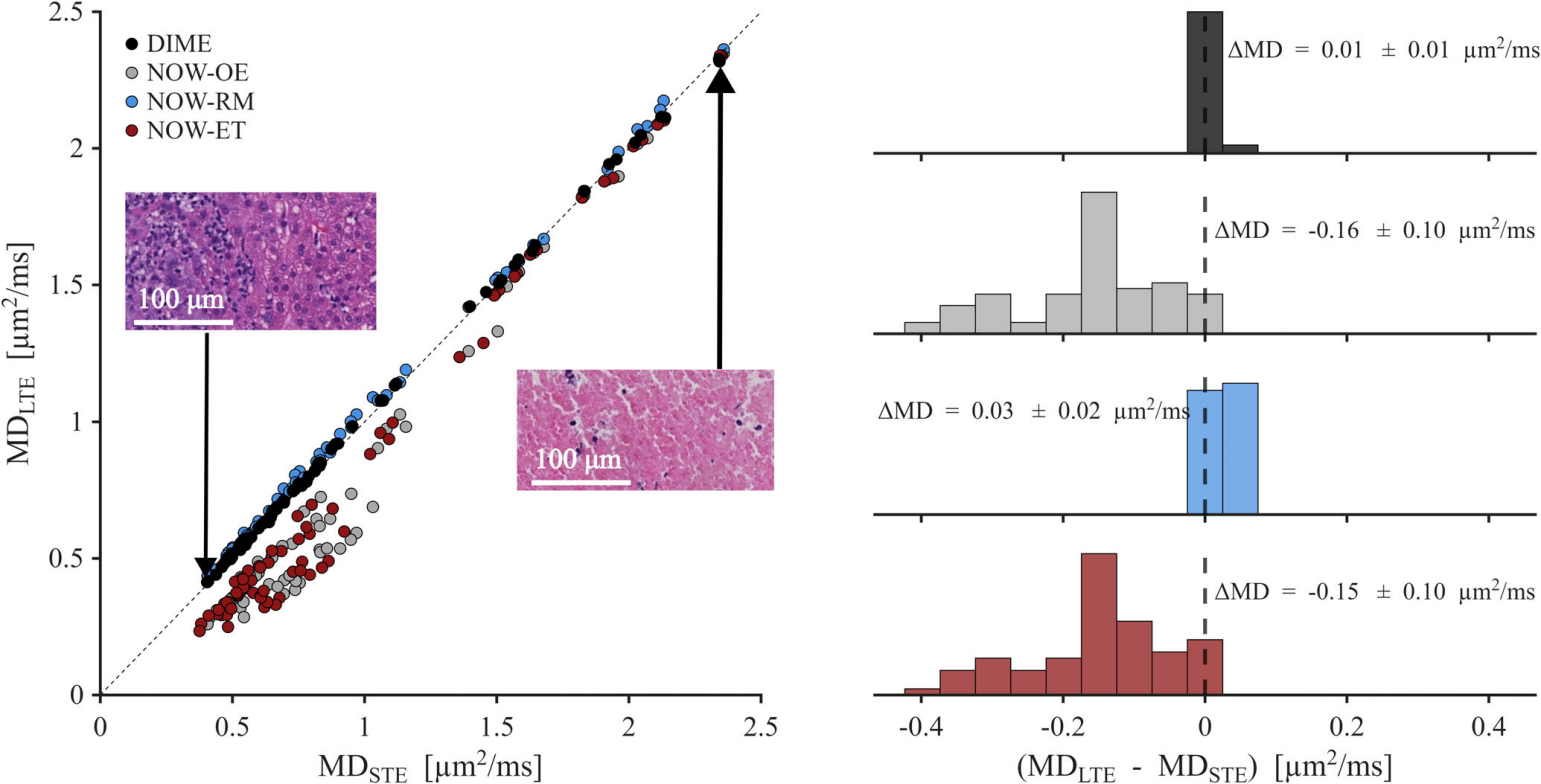
DIME produces less biased estimates in realistic histology‐based distributions compared to the NOW waveforms in both the 80 mT/m and 200 mT/m systems. DIME exhibited a narrow distribution of ΔMD centered on $0\;\mu m^{2}/\text{ms}$. NOW‐OE and NOW‐ET exhibited wider distributions with marked positive or negative biases, whereas NOW‐RM is only slightly positively offset and substantially closer to DIME. Substrates span histology‐derived tissues from mixed colorectal carcinoma and normal liver (lowest MD) to hepatocellular carcinoma with necrosis (highest MD).

Finally, DIME had a lower encoding efficiency compared to the less constrained NOW variants. This trade‐off is shown in Figure 9, which reports the encoding duration required to reach $b=2\;\text{ms}/\mu m^{2}$ based on the efficiency values in Table 1. DIME produced matched STE and LTE efficiency, with minimal durations of 84 ms on the 80 mT/m system and 62 ms on the 200 mT/m system. Thus, compared to NOW, DIME required longer encoding durations $\tau_{\text{enc}}$ by 13–15 ms at 80 mT/m, and 18–22 ms at 200 mT/m. Note that this analysis considers the required durations for a given pair of STE and LTE waveforms.

**FIGURE 9:**
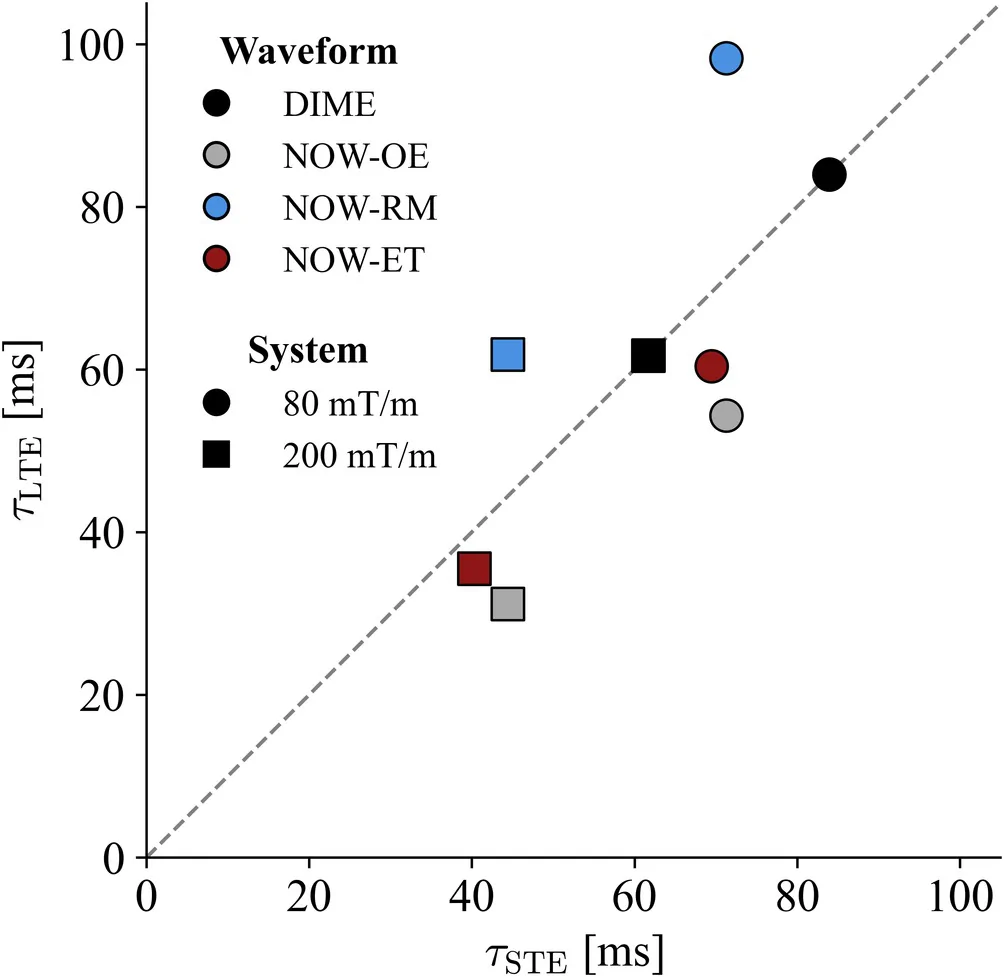
Encoding duration $\tau_{\text{enc}}$ required to reach $b=2\;\text{ms}/\mu m^{2}$ as implied by κ, for STE and LTE in DIME, the efficiency‐optimized NOW‐OE, restriction‐matched NOW‐RM, and encoding‐time‐matched NOW‐ET on the 80 (circles) and 200 mT/m (squares) gradient systems. The dashed identity line indicates equal STE and LTE efficiency. Points closer to the origin are more efficient overall. Overall, DIME is less efficient than NOW variants, requiring between 13 to 22 ms longer encoding duration compared to NOW. This duration is derived from κ for a target b‐value and was not the duration used during optimization.

## 5 Discussion

The present work proposed DIME to address four challenges related to tensor‐valued diffusion encoding in the presence of time‐dependent diffusion. First, DIME reduces the effects of anisotropic restrictions by enforcing that both diffusion and restriction weighting are isotropic. Second, it reduces the effects of differences in overall restriction sensitivity across waveforms with linear and spherical b‐tensor shapes. The benefits of these design features were consistent across a wide range of cylindrical, spherical, and histology‐derived substrates. Third, concomitant gradient balance was satisfied by exploiting gradient waveform symmetries and k‐nulling to suppress the effect of concomitant dephasing. Finally, peripheral and cardiac nerve stimulation levels were explicitly controlled in the numerical optimization to ensure that waveforms comply with safety limits. Taken together, DIME enables more accurate measurements and quantification when employing models and representations that assume consistent restriction sensitivity across waveforms [9, 25, 26], and the optimization framework facilitates arbitrary hardware constraints, nerve stimulation, and accurate gradient balance.

DIME controls orientation dependence by enforcing spectral isotropy across the encoding power spectrum as quantified by the m‐tensor (Figure 6). The potentially confounding effects of this orientation dependence were first recognized by de Swiet and Mitra [25], who identified two distinct effects of restricted diffusion on single‐shot trace measurements: an orientation dependence caused by unequal weighting of the diffusion spectrum along different directions (i.e., due to spectral anisotropy), which persists even when the b‐tensor is perfectly spherical [26]; and a difference in the measured diffusivity when the encoding does not probe the long‐time limit. DIME addresses the first confounder by enforcing isotropic diffusion‐ and restriction weighting (Equation (13)), ensuring isotropic low‐frequency restriction sensitivity.

DIME matches the apparent diffusion coefficients across waveforms by enforcing spectral matching as quantified by m/b (Figures 7 and 8). This addresses the second confounder identified by de Swiet and Mitra [25], regardless of b‐tensor shape. In tensor‐valued diffusion encoding, the hallmark of this confounder is that the signal encoded with different waveforms diverges already at low b‐values [24]. Although the effect can be difficult to detect, it may cause a large divergence at high b‐values, which is misinterpreted as microscopic diffusion anisotropy [23, 24, 27]. DIME waveforms are designed to solve this by constructing a matched LTE from the STE waveform. Since the STE has isotropic b‐ and m‐tensors, any projection onto a vector u inherits the same m/b (Equation (18)), ensuring that STE and LTE share the same leading‐order low‐frequency restriction sensitivity (Equation (14)). Moreover, specific projections allow for the two waveforms to be equally efficient, such that the pair can always produce the same b‐value for a given gradient amplitude and encoding duration (Figure 9). Recently, the SPAS approach by Lasič et al. (2025) addressed the same problem through so‐called ‘spectral tuning’ [23], offering two strategies: a projection‐based approach that selects the LTE direction whose apparent diffusivity matches the MD of the STE for an assumed diffusion model, and a geometric averaging approach that achieves a similar tuning by combining signals from multiple LTE waveforms. DIME differs from these because it enforces matching via waveform design, ensuring that every individual LTE direction maintains the same m/b as the STE by construction. This removes the implementation‐dependence noted by Hamilton et al. [28], since it does not rely on any specific tuning strategy. We note, however, that the DIME framework can be modified to accept alternative restriction weighting parameters in place of the m‐tensor; however, exploring such options was outside the scope of this work.

The appeal of using the m‐tensor is its simplicity; however, it only completely removes spectral anisotropy if the restriction effects follow the low‐frequency approximation of D(ω) for closed compartments, which is not the general case. Nevertheless, our investigation showed that DIME outperformed NOW in all tested substrates, although it was not perfect. Thus, we expect the best performance for relatively small closed compartments, where restriction effects scale as $\omega^{2}$ [32, 52], but the benefits extend also beyond this special case. As emphasized by Nilsson et al. [32], we expect a degrading performance as more encoding power is placed above the lowest transition frequency of the compartment (Supporting Information, Equation (C9)), beyond which D(ω) differs markedly from the $\omega^{2}$‐behavior. In simulations of cylinders, the CV of DIME remained below 1% up to 5–6 μm radius on both systems (Figure 6). Other structural environments have been shown to give rise to different spectral scaling behaviors, including $\omega^{1/2}$ for diffusion restricted by randomly placed permeable membranes [30] and $\omega^{1}$ for hindered extracellular diffusion in disordered axonal packings [31, 53]. Hence, matching m/b should not be interpreted as full spectral identity between STE and LTE, and isotropy of M may fail to eliminate directional differences in restriction sensitivity. Despite this drawback, we note that the choice of exponent used in waveform optimization may not be critical to the performance. Although we constrained only the shape of the b‐ and m‐tensors (p=0 and 2, Equation (5)), DIME waveforms had markedly lower anisotropy and closer matching compared to NOW for corresponding objects with 0<p<2 (Figure S2). The constraints used in DIME therefore confer their isotropic characteristics, at least partially, to corresponding objects of intermediate order, which likely explains the high performance of DIME across the tested substrates. Optimizing with constraints on other orders of p would require abandoning the fast analytic constraint formulation, and remains an avenue for future work.

Both DIME and the restriction‐matched NOW‐RM exhibited excellent matching in ideal and realistic substrates (Figures 7 and 8). This indicates that the restriction matching method proposed herein (Equation (14)) is viable and provides a straightforward method to derive matched LTE from STE waveforms that are already in use. Despite an identical matching strategy, DIME consistently outperformed NOW‐RM which suggests that the DIME waveform design has additional benefits. We speculate that this is due to the use of a triplet of encoding pulse pairs, which affords additional similarities across axes that are not controlled or captured by the m‐tensor.

The simulations in cylinders and spheres isolated specific mechanisms and facilitated the comparison with NOW under challenging conditions. A single, coherently oriented cylinder constitutes the worst case for rotational variance. It combines maximal diffusion anisotropy with the absence of the orientation dispersion that would otherwise average out directional sensitivity [32]. Spherical substrates remove anisotropy entirely, so that any STE and LTE difference is attributable to restriction weighting alone [23, 24], isolating the matching problem. Pairing analytical solutions for restricted diffusion in cylinders and spheres with Monte Carlo simulations provides mutually independent references, since the analytical signals are free of the sampling, discretizations, and step‐size sensitivities that limit numerical approaches [54]. Histology‐derived substrates then probe whether the effects survive in heterogeneous, tissue‐like geometries [51]. The choice of substrates ensures that the observed advantage of DIME can be attributed to controlled mechanisms rather than a single favorable geometry.

The histology‐derived substrates provide more realistic substrates and incorporate tissue‐like heterogeneity with contributions from the intra‐ and extracellular space. These substrates therefore provide an intermediate level of validation between controlled analytical geometries and in vivo measurements. However, they are a simplification; presently, the framework is based on two‐dimensional histological sections and does not fully capture three‐dimensional tissue organization, exchange [51, 52, 54, 55], relaxation, perfusion, or experimental imperfections. Although simulations support the conclusion that the benefits of DIME are not limited to a single idealized geometry, further validation in larger, three‐dimensional, exchange‐enabled substrates, and ultimately in vivo, is warranted to map the validity of DIME and its possible variants.

Finally, we note two additional limitations. First, the use of three pairs of trapezoids enables a parsimonious description of the waveform, but it also limits the optimization search space and reduces the encoding efficiency compared to optimizing arbitrary number of control points [39]. For the tested configurations, the additional encoding duration ranged between 13 and 22 ms (Figure 9), where the majority of the added encoding duration can be attributed to the waveform design (Supporting Information, Section E). Thus, the improved measurement accuracy of DIME must be weighed against reduced signal‐to‐noise ratios imposed by longer echo times, and potentially longer acquisition times. Recently, two studies on mouse brains and prostate cancer xenografts demonstrated that mismatched restriction sensitivity across waveforms causes severe bias in diffusion parameters [23, 27], but the size of the effect on similar studies performed on clinical MRI systems is still a topic of active investigation. Second, the validity of k‐nulling is known to degrade if the gradients are nonuniform [56]. Implementations on systems with pronounced nonuniformity should therefore verify that the suppression of concomitant gradient effects is sufficient to yield accurate signal.

## 6 Conclusion

DIME provides sets of waveforms for STE and LTE with matched restriction weighting both within and between gradient waveforms. This can improve the reliability and accuracy of quantification based on tensor‐valued diffusion encoding in the presence of restriction effects. DIME also explicitly accounts for MRI hardware restrictions, gradient balance including concomitant gradients, as well as patient safety in terms of both peripheral and cardiac nerve stimulation. Compared with state‐of‐the‐art NOWs, DIME offers a practical and deployable framework for more reliable tensor‐valued diffusion encoding in settings where time‐dependent diffusion cannot be ignored.

## Funding

This work was supported by eSSENCE@LU (Grant No. 12:3), the Swedish Prostate Cancer Federation (Grant No. 2025‐02‐21), Crafoordska Stiftelsen (Grant No. 20240791, http://dx.doi.org/10.13039/501100003173), Cancerfonden (Grant No. 22 0592 JIA, http://dx.doi.org/10.13039/501100002794), and Vetenskapsrådet (Grant No. 2021‐04844, http://dx.doi.org/10.13039/501100004359).

## Conflicts of Interest

Authors S.L. and F.S. are inventors of related IP and may financially benefit from its use. All other authors declare no commercial or financial relationships that could be viewed as a potential conflict of interest in relation to this research.

## Supporting information




**Figure S1:** STE and LTE waveforms for the 80 mT/m system and their encoding power spectra. The top row shows the three STE gradient components, the middle row the corresponding LTE waveform, and the bottom row the resulting encoding power spectra. As in the 200 mT/m system shown in Figure 4, double‐isotropic matched encoding (DIME) yields similar spectral shapes across the STE axes and close agreement between the STE and LTE spectra, consistent with matched restriction sensitivity. By contrast, the NOW‐based waveforms show greater axis‐to‐axis spectral variation and poorer agreement between STE and LTE, particularly for NOW‐OE and NOW‐ET.
**Figure S2:** Encoding metrics as a function of the spectral order *p*. **(A)** Ratio of the restriction weighting $m^{\left(p\right)}/b$ of the LTE to that of the STE in each waveform. A value of unity means the pair is matched in restriction weighting at that order. **(B)** Normalized anisotropy $\zeta_{m}^{p}$ of the STE waveforms. Both quantities are fixed by construction at p=0 and p=2 for DIME, and at p=0 only for NOW‐OE. Solid and dashed lines denote the 200 and 80 mT/m systems, respectively.
**Figure S3:** Excess encoding duration by enforcing an m‐tensor isotropy constraint, plotted against the achieved m‐tensor anisotropy at 80 and 200 mT/m. Duration is referenced to the original optimization tolerance constraint $\sqrt{\zeta_{m}}<0.01/3$ (left dotted line); the right dotted line marks the anisotropy of the NOW‐OE STE. Filled circles mark the fully unconstrained optimization. Less than 5 ms is recovered even without any constraint on **M**.

## Data Availability

The DIME optimizer, simulation code, and materials associated of this publication are available in open source at http://github.com/felixmortensen/DIME. The DIME optimizer is also distributed as part of the free gradient waveform toolbox available at http://github.com/filip-szczepankiewicz/fwf_sequence_tools.
