# Supplementary material for: Optimized Gradient Waveforms for Tensor‐Valued Diffusion MRI Under Time‐Dependent Diffusion Using Double‐Isotropic Matched Encoding (DIME)
Source: Magn Reson Med. 2026 Sep 24;97(1):225–39. doi: 10.1002/mrm.70602 (PMC13746097; doi:10.1002/mrm.70602)
Supplement: Supplementary file 1 [file MRM-97-225-s001.pdf]

Supporting Information

immediate

A Waveforms at 80 mT/m

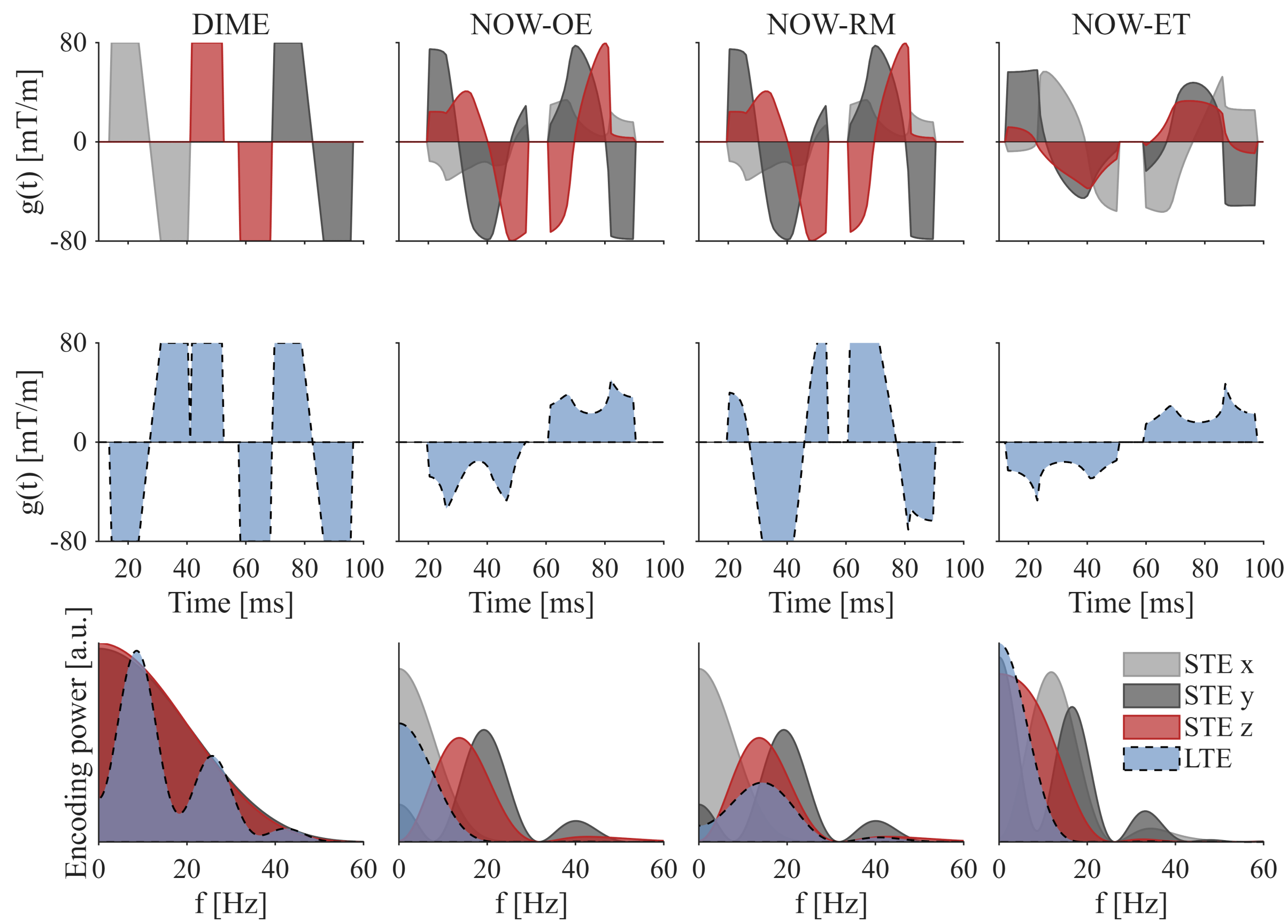

Figure S1 STE and LTE waveforms for the 80 mT/m system and their encoding power spectra. The top row shows the three STE gradient components, the middle row the corresponding LTE waveform, and the bottom row the resulting encoding power spectra. As in the 200 mT/m system shown in Figure 4, DIME yields similar spectral shapes across the STE axes and close agreement between the STE and LTE spectra, consistent with matched restriction sensitivity. By contrast, the NOW-based waveforms show greater axis-to-axis spectral variation and poorer agreement between STE and LTE, particularly for NOW-OE and NOW-ET.

B Closed-form expressions for the b- and m-values

Because the DIME waveform family is built from non-overlapping trapezoidal pulse pairs, the diagonal entries of the diffusion-weighting tensor **B** and restriction-weighting tensor **M** can be evaluated analytically. This avoids numerical integration of sampled waveforms during optimization and allows fast evaluation of the scalar weights $b = \sum_i B_{ii}$ and $m = \sum_i M_{ii}$.

For a one-dimensional gradient waveform $g(t)$, the corresponding dephasing trajectory is described by Equation 2 in the main text so that the one-dimensional diffusion-weighting factor is according to Equation 6. Likewise, in the low-frequency approximation, the corresponding one-dimensional relative restriction-weighting factor is according to Equation 7.

A trapezoidal pulse pair with gradient amplitude g, ramp-up time $t_\uparrow$, flat-top time t_{flat}, ramp-down time $t_\downarrow$, and pause time t_p gives the closed-form b-value

$$\begin{aligned} b(g, t_\uparrow, t_\downarrow, t_{\text{flat}}, t_p) = \gamma^2 g^2 \Bigg[& \frac{t_p \left(t_\downarrow + 2t_{\text{flat}} + t_\uparrow\right)^2}{4} \\ & + \frac{t_\downarrow}{30}\left(8t_\downarrow^2 + 40t_\downarrow t_{\text{flat}} + 20t_\downarrow t_\uparrow + 60t_{\text{flat}}^2 + 60t_{\text{flat}}t_\uparrow + 15t_\uparrow^2\right) \\ & + \frac{t_{\text{flat}}}{6}\left(4t_{\text{flat}}^2 + 6t_{\text{flat}}t_\uparrow + 3t_\uparrow^2\right) + \frac{t_\uparrow^3}{10}\Bigg]. \end{aligned} \tag{B1}$$

The corresponding m-value is

$$m(g, t_\uparrow, t_\downarrow, t_{\text{flat}}) = g^2 \left(\frac{2}{3}t_\uparrow + 2t_{\text{flat}} + \frac{2}{3}t_\downarrow\right). \tag{B2}$$

These expressions were applied separately to the xy- and z-components of the DIME waveform. By construction, the x- and y-components are identical, so $B_{xx} = B_{yy}$ and $M_{xx} = M_{yy}$, and all three diagonal elements of both tensors follow from the same trapezoidal formulas; the only structural difference being that the central z-pair includes a pause t_p around the refocusing interval, whereas the outer xy-pairs do not. Since the waveform family keeps **B** and **M** diagonal, the scalar weightings are simply the traces, such that

$$b = \sum_{i=1}^{3} B_{ii} = B_{xx} + B_{yy} + B_{zz}, \qquad m = \sum_{i=1}^{3} M_{ii} = M_{xx} + M_{yy} + M_{zz}. \tag{B3}$$

C Analytical solutions to the diffusion spectrum for cylinders and spheres

For the analytical signal simulations in the idealized substrates, the diffusion spectra were evaluated in the frequency domain using closed-form expressions for restricted diffusion in simple geometries as described by [1, 2, 3]. Along a principal axis of a restricted geometry, the diffusion spectrum can be written as a weighted sum of Lorentzian terms,

$$D_{\mathrm{res}}^{(d)}(\omega) = D_0 \sum_{k=1}^{\infty} \frac{a_k B_k \omega^2}{a_k^2 D_0^2 + \omega^2}, \tag{C1}$$

where D_0 is the free diffusivity and d specifies the geometry. In compact form, the coefficients are

$$a_k = \left(\frac{z_k}{R}\right)^2, \qquad B_k = \frac{2}{z_k^2 + 1 - d}\, a_k^{-1}, \tag{C2}$$

where R is the restriction radius and z_k are the positive roots of

$$z J_{d/2-1}(z) - (d-1) J_{d/2}(z) = 0, \tag{C3}$$

with J_ν denoting the Bessel function of the first kind of order ν.

For an infinitely long cylinder aligned with the z-axis, diffusion is unrestricted along the cylinder axis and restricted in the transverse plane. In this case, the cylindrical coefficients can be written explicitly as

$$a_k = \left(\frac{\mu_k}{R}\right)^2, \qquad B_k = \frac{2(R/\mu_k)^2}{\mu_k^2 - 1}, \tag{C4}$$

where μ_k are the positive roots of

$$J_1'(\mu_k) = 0. \tag{C5}$$

The transverse diffusion spectrum is therefore

$$D_\perp(\omega) = D_0 \sum_{k=1}^{\infty} \frac{a_k B_k \omega^2}{a_k^2 D_0^2 + \omega^2}, \tag{C6}$$

and the full tensor becomes

$$\mathbf{D}_{\mathrm{cyl}}(\omega) = \mathrm{diag}(D_\perp(\omega), D_\perp(\omega), D_0)\,. \tag{C7}$$

Equivalently, the cylindrical spectrum may be written in the form used by Nilsson et al. (2017) [2],

$$D_\perp(\omega) = D_0 - \sum_{k=1}^{\infty} \frac{C_k}{1 + (\omega/b_k)^2}, \tag{C8}$$

with

$$b_k = a_k D_0 = \frac{\mu_k^2 D_0}{R^2}, \qquad C_k = b_k B_k = \frac{2D_0}{\mu_k^2 - 1}. \tag{C9}$$

For a sphere, the restriction is isotropic, and the diffusion spectrum is therefore

$$\mathbf{D}_{\mathrm{sph}}(\omega) = D_{\mathrm{res}}^{(3)}(\omega)\,\mathbf{I}, \tag{C10}$$

where $D_{\mathrm{res}}^{(3)}(\omega)$ is obtained from Equation (C1) by setting $d = 3$.

These spectra were inserted into the frequency-domain signal expression of Equation 1 to generate the analytical signals used for the cylinder and sphere simulations. In practice, the infinite series in Equation (C1) were truncated after 60 terms to ensure numerical convergence over the frequency range spanned by the encoding waveforms.

D Restriction weighting and anisotropy at arbitrary spectral order p

DIME constrains the encoding at a quadratic frequency dependence of $D(\omega)$ ($p = 2$ in Equation (3)), whereas other structural universality classes give other exponents.

The scalar moment m_p of Equation (5) generalizes to a tensor in the same way that b and m generalize to $\mathbf{B}$ and $\mathbf{M}$,

$$M_{ij}^{(p)} = \frac{1}{2\pi}\int_{-\infty}^{\infty} |\omega|^p \,\mathrm{Re}\big[Q_i(\omega)\,Q_j^*(\omega)\big]\;\mathrm{d}\omega. \tag{D1}$$

The moments were evaluated from the effective gradient waveform at $N_s = 2730$ samples of duration $\Delta t = 46$ µs. The dephasing vector was obtained by cumulative trapezoidal integration of Equation (2), and its spectrum from a zero-padded discrete Fourier transform,

$$Q_i(\omega_k) = \Delta t \sum_{n=0}^{N-1} q_{i,n}\, e^{-\mathrm{i}\omega_k n \Delta t}, \qquad \omega_k = \frac{2\pi k}{N\Delta t}, \tag{D2}$$

where $N = PN_s$ with a zero-padding factor $P = 16$, and ω_k is mapped onto the signed interval $(-\pi/\Delta t,\, \pi/\Delta t]$. Equation (D1) then becomes a Riemann sum in which the factors $1/2\pi$ and $\Delta\omega$ collapse into a single prefactor,

$$M_{ij}^{(p)} \simeq \frac{1}{N\Delta t}\sum_{k=0}^{N-1} |\omega_k|^p \,\mathrm{Re}\big[Q_i(\omega_k)\,Q_j^*(\omega_k)\big]\,. \tag{D3}$$

The implementation was verified at the two orders where a closed form exists: $\mathbf{M}_0$ and $\mathbf{M}_2$ from Equation (D3) agreed with $\mathbf{B}$ and $\mathbf{M}$ evaluated directly in the time domain from Equations (8) and (9).

Figure S2 shows the restriction weighting ratio $m_{\mathrm{LTE}}/m_{\mathrm{STE}}$ and the anisotropy ζ_m across $0 \leq p \leq 2$. The DIME STE/LTE pair remains matched to within about 1% of unity at every order, whereas the NOW-OE matching deteriorates monotonically as p increases. The anisotropy optimized for $\sqrt{\zeta_m} < 0.01/3 \Rightarrow \zeta_m < 0.0001$ at both endpoints for DIME, and peaks at $\zeta_m \approx 0.01$ for $p \approx 0.75$, while NOW-OE increases monotonically to $\zeta_m \approx 0.20$. Constraining the m-tensor shape at both $p = 0$ and $p = 2$ partially confirs its isotropy to intermediate powers p.

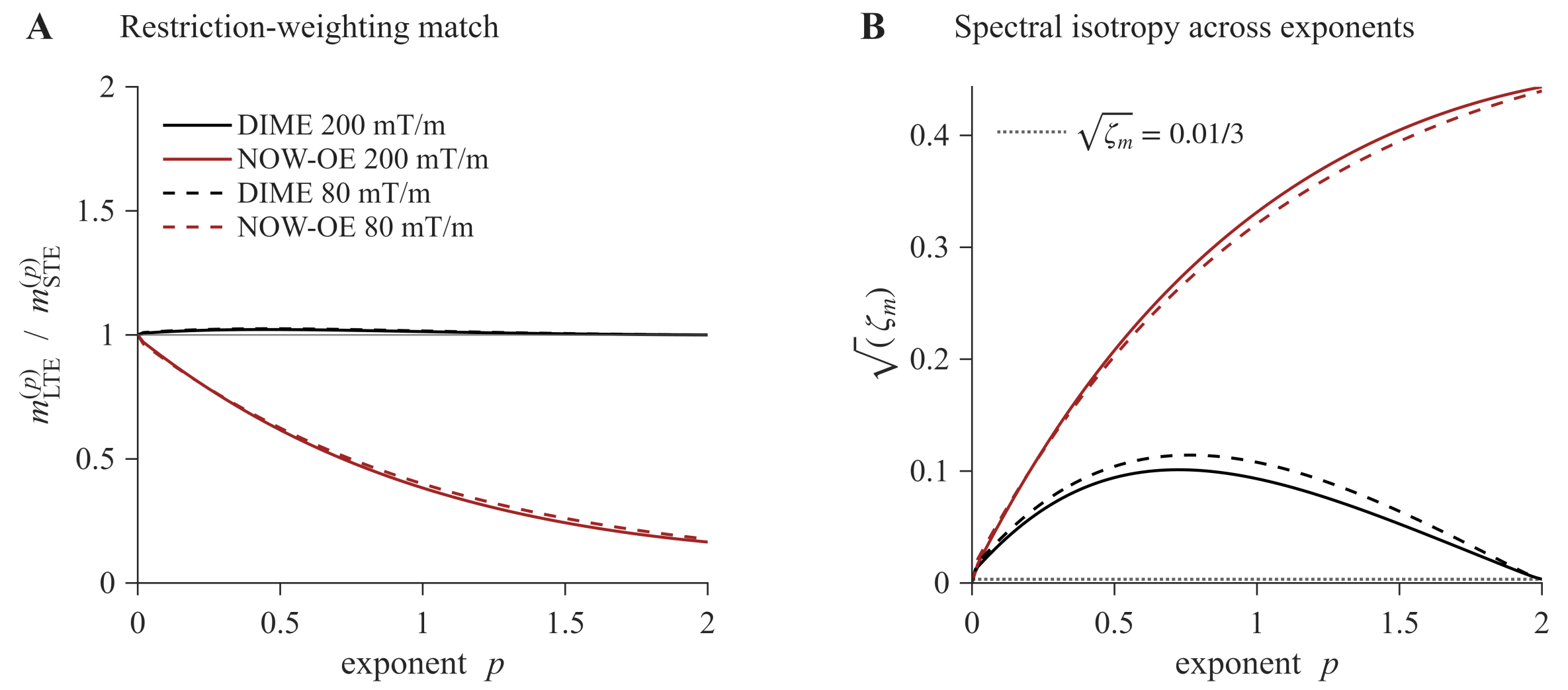

Figure S2 Encoding metrics as a function of the spectral order p. **(A)** Ratio of the restriction weighting $m^{(p)}/b$ of the LTE to that of the STE in each waveform. A value of unity means the pair is matched in restriction weighting at that order. **(B)** Normalized anisotropy $\zeta_m^{(p)}$ of the STE waveforms. Both quantities are fixed by construction at $p = 0$ and $p = 2$ for DIME, and at $p = 0$ only for NOW-OE. Solid and dashed lines denote the 200 and 80 mT/m systems, respectively.

E Encoding duration penalty from m-tensor anisotropy

To quantify how much encoding duration is spent enforcing exact isotropy of the m-tensor, the isotropy tolerance imposed during optimization was swept over $3\sqrt{\zeta_m} <$ [0.01, 0.02 , 0.05, 0.10, 0.20, 0.50, unconstrained], with the b-tensor isotropy tolerance held fixed at 0.01 throughout so that only the m-tensor was allowed to relax. At each tolerance, the resulting m-tensor anisotropy ζ_m and the encoding duration τ_{enc} required to reach $b = 2$ ms/µm^2 was computed, taking the best of repeated optimizations run from both directions of the tolerance sweep, which avoids biasing solutions toward the more constrained tolerance and understating the recoverable duration. Figure S3 shows the resulting encoding duration lost relative to the tolerance used in the main text ($\sqrt{\zeta_m} < 0.01/3$), as a function of the anisotropy ζ_m. Relaxing the tolerance to 0.05 recovers just 0.8 ms and 0.9 ms of encoding duration on the 80 and 200 mT/m systems, respectively (about 1%), and removing the constraint entirely recovers only 3 ms (about 3%) and 5 ms. Even with no constraint at

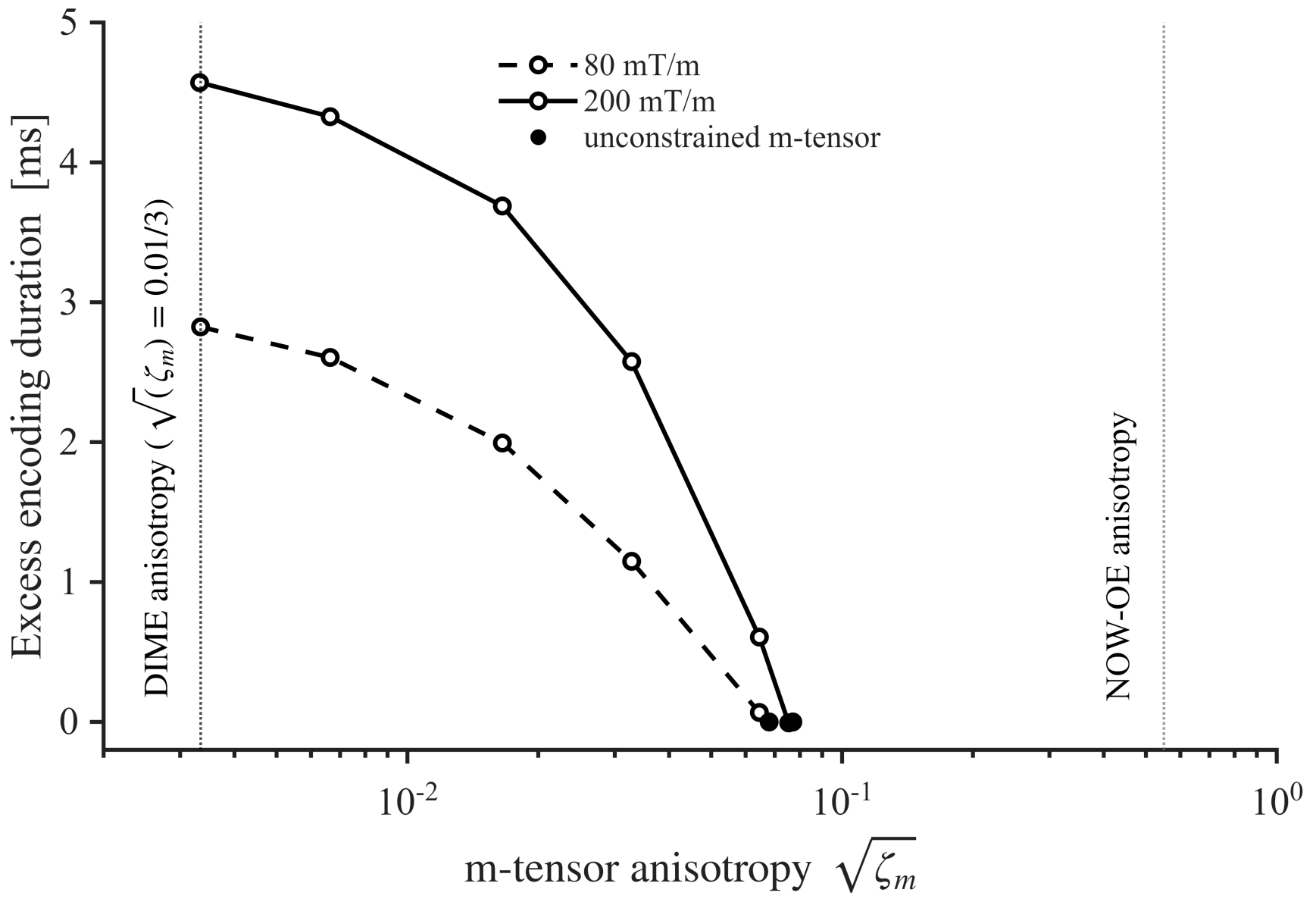

Figure S3 Excess encoding duration by enforcing m-tensor isotropy constraint, plotted against the achieved m-tensor anisotropy at 80 and 200 mT/m. Duration is referenced to the original optimization tolerance constraint $\sqrt{\zeta_m} < 0.01/3$ (left dotted line); the right dotted line marks the anisotropy of the NOW-OE STE. Filled circles mark the fully unconstrained optimization. Less than 5 ms is recovered even without any constraint on **M**.

all the waveform achieves an anisotropy of $\zeta_m \approx 0.07$ rather than a fully anisotropic solution, far below the $\zeta_m \approx 0.2$ of the NOW-OE STE it is compared against. The orthogonal, self-refocused block design already favors an isotropic m-tensor on its own. The isotropy constraint used in the manuscript therefore converts this near-isotropy into exact isotropy at a cost of only a few milliseconds of encoding duration, and the main encoding duration penalty comes from the parametrization of the trapezoidal design itself.

References

[1] Janez Stepišnik. "Time-dependent self-diffusion by NMR spin-echo". In: *Physica B: Condensed Matter* 183.4 (May 1, 1993), pp. 343–350. ISSN: 0921-4526. DOI: 10.1016/0921-4526(93)90124-O. URL: https://www.sciencedirect.com/science/article/pii/092145269390124O.

[2] Markus Nilsson et al. "Resolution limit of cylinder diameter estimation by diffusion MRI: The impact of gradient waveform and orientation dispersion". In: *Nmr in Biomedicine* 30.7 (July 2017), e3711. ISSN: 0952-3480. DOI: 10.1002/nbm.3711. URL: https://www.ncbi.nlm.nih.gov/pmc/articles/PMC5485041/.

[3] Henrik Lundell and Samo Lasic. "Chapter 2. Diffusion Encoding with General Gradient Waveforms". In: *New Developments in NMR*. Ed. by Daniel Topgaard. Cambridge: Royal Society of Chemistry, 2020, pp. 12–67. ISBN: 978-1-78801-726-8. DOI: 10.1039/9781788019910-00012. URL: http://ebook.rsc.org/?DOI=10.1039/9781788019910-00012.
